# Supplementary material for: The structure and robustness of ecological networks with two interaction types
Source: PLoS Comput Biol. 2024 Jan 19;20(1):e1011770. doi: 10.1371/journal.pcbi.1011770 (PMC10830016; doi:10.1371/journal.pcbi.1011770)
Supplement: S1 Text — (PDF) [file pcbi.1011770.s001.pdf]

SUPPLEMENTARY INFORMATION FOR:
THE STRUCTURE AND ROBUSTNESS OF ECOLOGICAL NETWORKS WITH TWO INTERACTION TYPES

A PREPRINT

Virginia Domínguez-García, ORCID 0000-0002-4591-4186
ISEM, CNRS, Univ. Montpellier, IRD, EPHE, Montpellier, France
Estación Biológica de Doñana (EBD-CSIC), Sevilla, Spain;
virginia.dominguez@ebd.csic.es

Sonia Kéfi, ORCID 0000-0002-9678-7770
ISEM, CNRS, Univ. Montpellier, IRD, EPHE, Montpellier, France;
Santa Fe Institute, Santa Fe, New Mexico, United States of America
sonia.kefi@umontpellier.fr

January 13, 2024

Supporting information Text

1 Data-set compilation

In order to build the data-set of tripartite networks, we gathered networks from 6 different studies (see Fig A) containing networks with two or more different types of interactions. When a study included networks from many sites and habitats, we considered all the individual networks as well as the merged versions (e.g. in the Macfayden study [1], we considered the networks of the different organic or traditional farms, and also the two networks merging all the traditional and all the organic farms). When a study contained networks with more than two interactions, we considered all the tripartite networks (composed by only two different interactions) that could be generated combining all the interaction layers in the original study. From all the rendered networks we only considered those with at least 5 species in the shared set, resulting in the 44 networks that form our data set (see Table 2 in the main text). The goal was to obtain as many different networks as possible from each reference, circumventing the problem of having too few linking nodes to carry out statistical calculations. Note that the results shown in the manuscript hold also when removing networks that may contain redundant information. The name of the 44 networks in our data set includes first the name of the leading author of the original study (e.g. McFayden) followed by the number of the site or '00' if there was only one site in the original study, a code describing the habitat type (e.g 'A' or 'B' for the two different types of farms in the study) or '00' if the original study only included one habitat type, and a code describing the two interactions included in the network (e.g. PH for pollination-herbivory, SDH for seed dispersal-herbivory, HPa for herbivory-parasitism, PSD for pollination-seed dispersal and PA for pollination-ant mutualism) (see Table 2 in main text).

2 Heterogeneous degree distributions and disassortativity

We measure basic structural features (degree heterogeneity and degree-degree correlations) in the tripartite networks in our data set. We find that they have a highly heterogeneous degree distribution, with an average value of $\sigma_k/{<}k{>}$ of 1.26 (see Fig BA). Grouping networks according to the sign of the two ecological interactions involved, we find small differences among them, with MA networks being slightly more heterogeneous than AA networks (see Fig BA), but the differences are not significant. Comparing the degree heterogeneity of the networks to that measured in null model "1" random ensemble (where the number of nodes in each species set and the number of links in each interaction layer are kept constant) shows that all the observed networks have a degree distribution significantly more heterogeneous than the null expectation, with Z-Score values over 1.96 in all networks but one, independent of the interaction type (see Fig BC and Table A below).

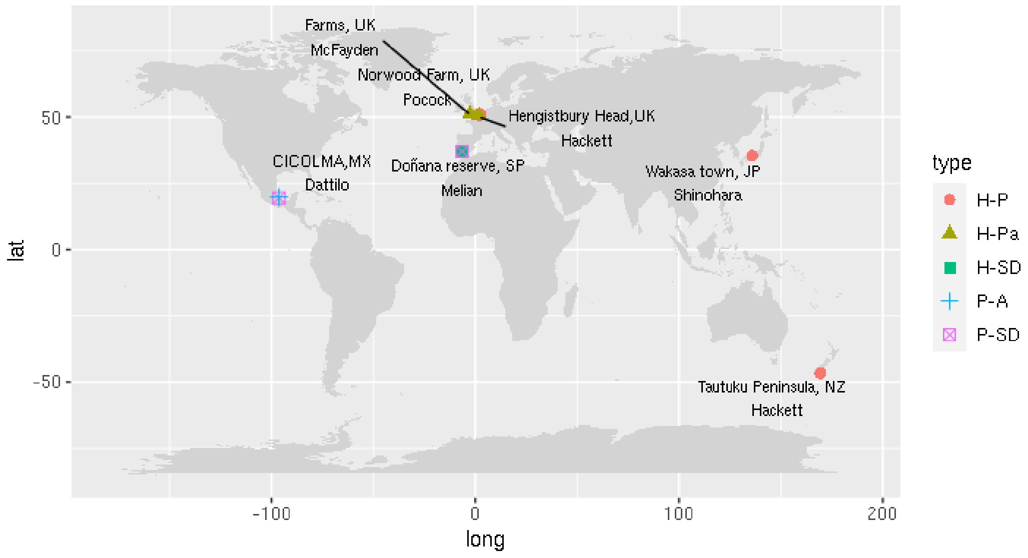

Fig A: Map showing the location of the 6 studies we used to build the database of networks. The color and shape of the points represents the different types of networks, and the text the name of the site, the country, and the surname of the leading author of the study. The map has been done by the authors using package "rnaturalearth"[2] in R[3]

.

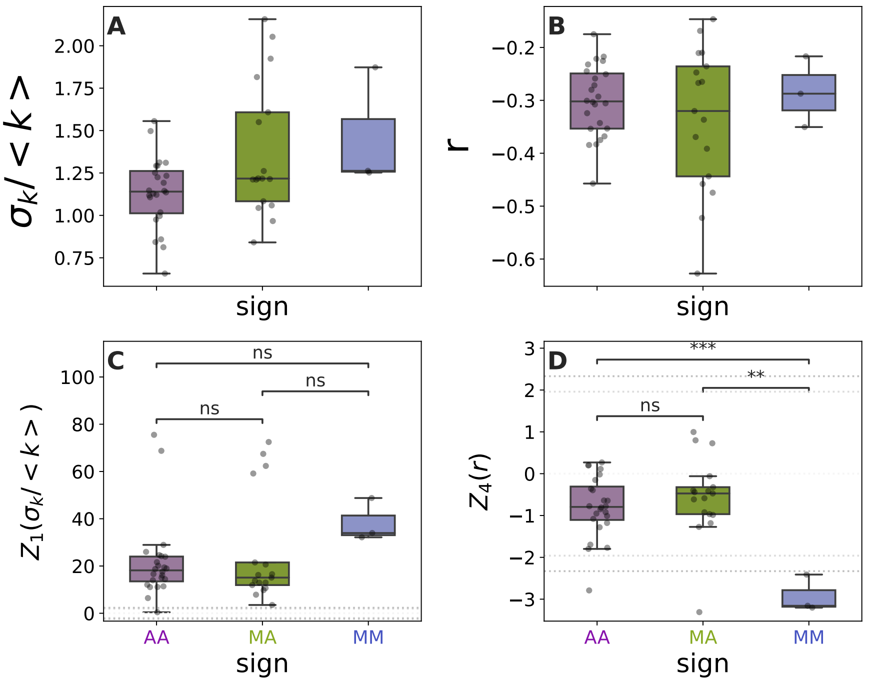

Fig B: Boxplots of the basic structural features in the empirical tripartite networks of our data-set grouped (and color coded) by the sign of the interactions involved. A: Degree Heterogeneity ($\sigma_k / < k >$) B: Degree-degree correlations (r). C: Z-score of the degree heterogeneity ($\sigma_k / < k >$) in null model "1". D: Z-score of the degree-degrees correlations (r) in null model "4" in the tripartite networks. In B and D horizontal grey lines mark the limits of the confidence interval of 1.96 (95%) and 2.33 (98%). Differences between groups are measured by independent t-test (*** $p < 0.001$, ** $p < 0.01$, 'ns' not significant).

name	sign	int	Set a	N_a	Set b	N_b	Set shared	N_s	$\sigma_k/<k>$	r	$Z_1(\sigma_k/<k>)$	$Z_4(r)$	Ref.
Sinohara_1_ALL_PH	MA	H-P	Herbivore	26	Pollinator	23	Plant	49	1.08	-0.15	13.77(**)	0.73()	[4]
Sinohara_2_ALL_PH	MA	H-P	Herbivore	39	Pollinator	28	Plant	49	1.04	-0.21	12.96(**)	-0.59()	[4]
Sinohara_3_ALL_PH	MA	H-P	Herbivore	43	Pollinator	33	Plant	53	1.21	-0.25	16.53(**)	-0.42()	[4]
Sinohara_4_ALL_PH	MA	H-P	Herbivore	49	Pollinator	22	Plant	43	1.21	-0.37	11.93(**)	-3.31(**)	[4]
Sinohara_ALL_A_PH	MA	H-P	Herbivore	51	Pollinator	23	Plant	44	1.22	-0.27	12.94(**)	-0.97()	[4]
Sinohara_ALL_E_PH	MA	H-P	Herbivore	45	Pollinator	35	Plant	73	1.22	-0.21	21.51(**)	-0.32()	[4]
Sinohara_ALL_I_PH	MA	H-P	Herbivore	43	Pollinator	27	Plant	46	1.26	-0.24	16.21(**)	-0.47()	[4]
Sinohara_2_E_PH	MA	H-P	Pollinator	20	Herbivore	18	Plant	33	0.97	-0.26	7.86(**)	-1.18()	[4]
Sinohara_3_E_PH	MA	H-P	Pollinator	24	Herbivore	18	Plant	36	1.21	-0.32	15.10(**)	-1.27()	[4]
Sinohara_4_I_PH	MA	H-P	Herbivore	19	Pollinator	11	Plant	22	0.84	-0.17	3.51(**)	-0.06()	[4]
Melian_OO_OO_PH	MA	H-P	Pollinator	180	Herbivore	14	Plant	162	2.05	-0.44	72.48(**)	0.80()	[5]
Hackett_1_ALL_PH	MA	H-P	Pollinator	220	Herbivore	58	Plant	66	2.16	-0.39	62.36(**)	-0.40()	[6]
Hackett_2_ALL_PH	MA	H-P	Pollinator	53	Herbivore	25	Plant	40	1.06	-0.34	9.78(**)	-0.61()	[6]
Hackett_1_S_PH	MA	H-P	Pollinator	73	Herbivore	19	Plant	19	1.61	-0.63	10.67(**)	1.00()	[6]
Hackett_1_GL_PH	MA	H-P	Pollinator	86	Herbivore	19	Plant	31	1.55	-0.46	20.63(**)	-0.99()	[6]
Pocock_OO_OO_PH	MA	H-P	Pollinator	253	Herbivore	63	Plant	134	1.92	-0.47	67.46(**)	-0.44()	[7]
Melian_OO_OO_HSD	MA	H-SD	Herbivore	14	Seed disperser	27	Plant	188	1.82	-0.52	59.15(**)	-0.92()	[5]
McFayden_ALL_A_HPa	AA	H-Pa	Plant	113	Parasitoid	148	Host	393	1.56	-0.23	75.52(**)	-1.01()	[1]
McFayden_1_A_HPa	AA	H-Pa	Plant	25	Parasitoid	45	Host	110	1.31	-0.38	25.98(**)	-1.70()	[1]
McFayden_2_A_HPa	AA	H-Pa	Plant	31	Parasitoid	43	Host	84	1.31	-0.38	24.63(**)	-2.79(*)	[1]
McFayden_3_A_HPa	AA	H-Pa	Plant	33	Parasitoid	39	Host	107	1.25	-0.32	19.40(**)	-1.77()	[1]
McFayden_4_A_HPa	AA	H-Pa	Plant	38	Parasitoid	45	Host	92	1.02	-0.29	13.98(**)	-0.64()	[1]
McFayden_5_A_HPa	AA	H-Pa	Plant	41	Parasitoid	44	Host	107	1.12	-0.27	18.96(**)	-1.18()	[1]
McFayden_6_A_HPa	AA	H-Pa	Plant	32	Parasitoid	33	Host	82	1.14	-0.24	17.60(**)	-0.39()	[1]
McFayden_7_A_HPa	AA	H-Pa	Plant	35	Parasitoid	33	Host	90	1.14	-0.3	20.14(**)	-1.08()	[1]
McFayden_8_A_HPa	AA	H-Pa	Plant	35	Parasitoid	58	Host	101	1.23	-0.31	23.94(**)	0.20()	[1]
McFayden_9_A_HPa	AA	H-Pa	Plant	23	Parasitoid	28	Host	73	1.13	-0.35	16.61(**)	-0.92()	[1]
McFayden_10_A_HPa	AA	H-Pa	Plant	31	Parasitoid	43	Host	103	1.15	-0.35	15.06(**)	0.27()	[1]
McFayden_ALL_B_HPa	AA	H-Pa	Plant	95	Parasitoid	132	Host	333	1.5	-0.22	68.74(**)	-0.81()	[1]
McFayden_1_B_HPa	AA	H-Pa	Plant	33	Parasitoid	45	Host	114	1.29	-0.28	28.94(**)	-0.02()	[1]
McFayden_2_B_HPa	AA	H-Pa	Plant	31	Parasitoid	30	Host	62	0.98	-0.22	11.12(**)	-0.15()	[1]
McFayden_3_B_HPa	AA	H-Pa	Plant	35	Parasitoid	37	Host	93	1.29	-0.26	24.19(**)	-0.95()	[1]
McFayden_4_B_HPa	AA	H-Pa	Plant	28	Parasitoid	27	Host	72	1.12	-0.37	16.11(**)	-1.80()	[1]
McFayden_5_B_HPa	AA	H-Pa	Plant	25	Parasitoid	29	Host	63	1.19	-0.25	18.72(**)	-0.36()	[1]
McFayden_6_B_HPa	AA	H-Pa	Plant	30	Parasitoid	34	Host	81	0.84	-0.17	11.41(**)	0.11()	[1]
McFayden_7_B_HPa	AA	H-Pa	Plant	30	Parasitoid	39	Host	80	0.86	-0.23	12.07(**)	-0.85()	[1]
McFayden_8_B_HPa	AA	H-Pa	Plant	26	Parasitoid	36	Host	76	1.11	-0.38	14.43(**)	-1.28()	[1]
McFayden_9_B_HPa	AA	H-Pa	Plant	21	Parasitoid	26	Host	62	1.0	-0.3	11.07(**)	-0.78()	[1]
McFayden_10_B_HPa	AA	H-Pa	Plant	25	Parasitoid	29	Host	75	1.22	-0.34	21.62(**)	-0.78()	[1]
Hackett_1_ALL_HPa	AA	H-Pa	Plant	15	Parasitoid	17	Host	53	0.81	-0.31	6.43(**)	0.20()	[6]
Hackett_1_WL_HPa	AA	H-Pa	Plant	7	Parasitoid	7	Host	28	0.66	-0.46	0.43()	-0.65()	[6]
Melian_OO_OO_PSD	MM	P-SD	Pollinator	180	Seed disperser	27	Plant	82	1.87	-0.35	48.77(**)	-3.20(**)	[5]
Dattilo_OO_OO_PSD	MM	P-SD	Pollinator	173	Seed disperser	46	Plant	120	1.25	-0.29	32.15(**)	-3.16(**)	[8]
Dattilo_OO_OO_PA	MM	P-A	Pollinator	173	Ant	30	Plant	134	1.26	-0.22	33.96(**)	-2.41(*)	[8]

Table A: Tripartite empirical networks composing the data-set. Columns indicate from left to right: the name of the network (based on the publication it comes from), the sign of the two interactions composing the network (mutualism-mutualism: MM, antagonism-antagonism: AA, mutualism-antagonism: MA), the name of the two interactions layers composing the network (ant-mutualism: A, herbivory: H, parasitoidism: Pa, pollination: P, and seed-dispersal: SD), the degree heterogeneity ($\frac{\sigma_k}{<k>}$), the degree-degree correlations (r), Z-score of degree heterogeneity in the "NL" null model ($Z_{NL}\left(\frac{\sigma_k}{<k>}\right)$), Z-score of degree-degree correlations in null model "4" ($Z_4(r)$), and the reference to the study from which the data was gathered.

This is also true when looking at the average degree heterogeneity by species set (see Fig CA and C). This high degree heterogeneity means that empirical tripartite networks, contrary to the expectation in random networks where all nodes have a similar connectivity, have few nodes with a high connectivity (generalists) and many nodes with a lower connectivity (specialists), as shown in Fig D. This 'long tailed' degree distribution is a well established feature of plant-pollinator [9, 10], plant-seed disperser [9] and, to a lesser extent, food webs [11, 12] that we recover in the tripartite networks in our data-set as well. Finally, looking only at the degree heterogeneity of the nodes in the shared set, we find that in 50% of networks the degree heterogeneity of the nodes in shared set in the tripartite network is higher than the degree heterogeneity of the subset of shared set nodes in the two bipartite networks (Table B), and in all cases the degree heterogeneity of the shared set nodes in the tripartite is at least larger than that of one of the two subsets (Table B). This means that the two layers are connected in such a way that the degree heterogeneity of the shared set nodes is increased.

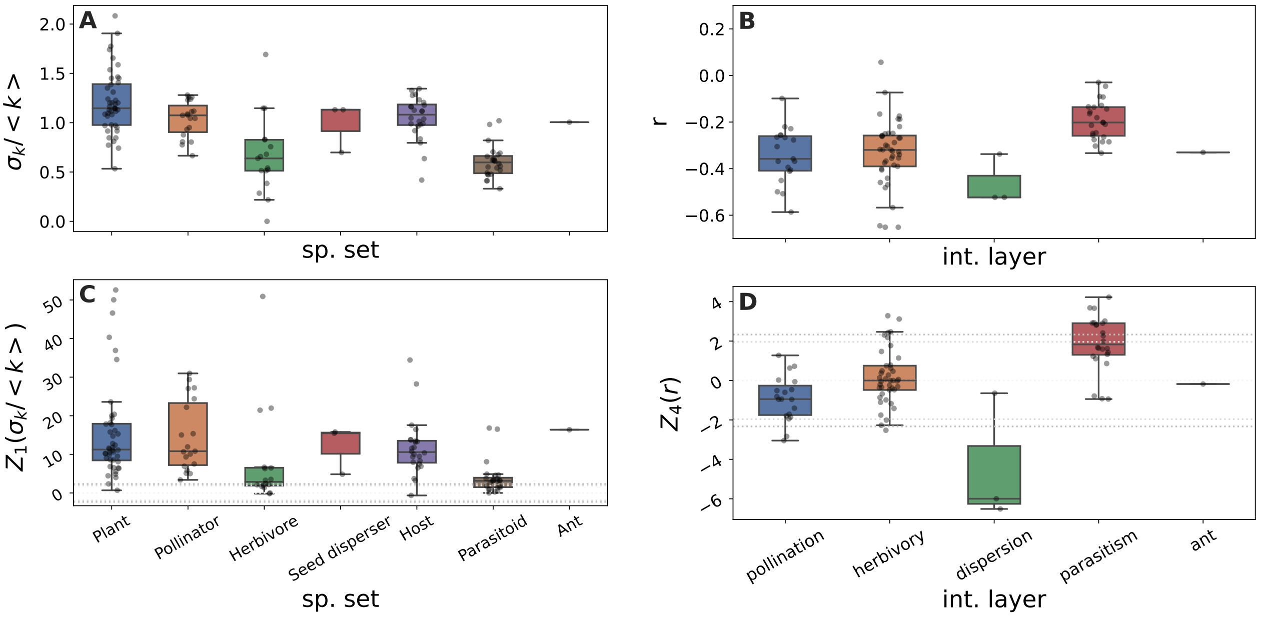

Fig C: A: Degree heterogeneity of the empirical tripartite ecological networks ($\sigma_k/<k>$) at the species set level B: Degree-degree correlations (r) at the interaction layer scale. C: Boxplot of the Z-score of the degree heterogeneity ($\sigma_k/<k>$) by species set in null model 1. D: Boxplot of the Z-score of the degree-degree correlations by interaction layer in null model 4. Horizontal grey lines mark the limits of the confidence interval of 1.96 (95%) and 2.33 (98%) for rejecting the null hypothesis.

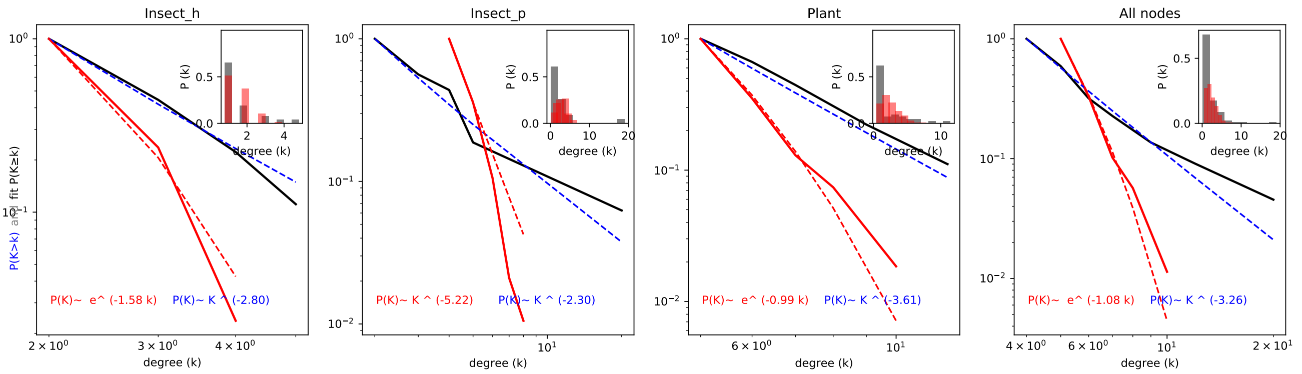

Fig D: Cumulative degree distribution in a multipartite 'mutualistic-antagonistic' network of pollination-herbivory compared with its randomization in null model 1. Figures show the degree distribution for the three different sets of species, from left to right: insect herbivores, insect pollinators, plants and all nodes combined (i.e. the merged network). The continuous black line is the empirical cumulative degree distribution, with the best power law distribution fit in a dashed blue line. The continuous red line is the cumulative degree distribution in null model "1" ensemble (100 randomizations), and the dashed red line the best exponential fit to that distribution. The insets show the degree distribution of the empirical network (in grey) and that of 100 randomizations (in red).

Apart from this heterogeneity in the species degree, in empirical networks the species are not randomly connected, rather the degree of a node can depend on the degree of its neighbours. This is known as 'mixing patterns' or degree-degree correlations, and is measured using correlation coefficients between the degree of a node and the degree of its neighbours. Pearson's correlation coefficient between the degree of a node and the average degree of the node's neighbours (r) was found to be negative in all empirical networks, indicating that they are all disassortative, i.e. that species with many connections tend to be linked to species with few connections (see Fig BB). Since degree heterogeneity is known to promote degree disassortativity [13], we also studied if the empirical networks were more disassortative than expected given their degree distribution by comparing the observed values to those of the constant "K" ensemble of null model 4. In line with previous findings in ecological plant-mutualistic networks [13], we find that most of the observed tripartite networks are not significantly more disassortative than the null expectation (with the exception of MM networks, which exhibit a higher disassortativity than expected), as the value of r in the empirical networks is consistent with those of the "K" ensemble average (null model 4) with a confidence con 95%(98%) in 86%(90%) of the networks (see Fig BD and Table A above).

When we look at the interaction layer scale (i.e. at the two unilayer networks composing the tripartite network), we find that all pollination, dispersion, ant-mutualism and a majority (80%) of herbivory networks are disassortative, while most of parasitoids networks (80%) are assortative (i.e. have $r > 0$), although most herbivory and parasitism networks exhibit weak degree-degree correlations (values of r close to 0), as shown in Fig CB. However, as with the tripartite networks, when we compare with the randomizations in null model 4 they tend to not be significantly more assorative/disassortative than the null expectation (Fig CD), with 75%(91%) or interaction layers having a value of r compatible with that of the random ensemble with a 95%(98%) confidence interval. This means that most of the assortativity/disassortativity found in the empirical networks stems from their low/high degree heterogeneity.

	name	layer_A	layer_B	N_A	N_B	LS_HD	lsA_HD	lsB_HD	Inc. LS HD
0	Sinohara_1_ALL_PH	herbivory	pollination	49	49	0.95	1.01	0.77	
1	Sinohara_2_ALL_PH	herbivory	pollination	63	53	0.97	0.63	0.93	*
2	Sinohara_3_ALL_PH	herbivory	pollination	72	57	1.15	1.00	0.80	*
3	Sinohara_4_ALL_PH	herbivory	pollination	75	39	1.20	1.05	1.04	*
4	Sinohara_ALL_A_PH	herbivory	pollination	80	38	1.23	1.33	0.66	
5	Sinohara_ALL_E_PH	herbivory	pollination	82	71	1.19	1.06	0.91	*
6	Sinohara_ALL_I_PH	herbivory	pollination	67	49	1.20	0.68	0.95	*
7	Sinohara_2_E_PH	pollination	herbivory	40	31	0.98	0.86	0.76	*
8	Sinohara_3_E_PH	pollination	herbivory	42	36	1.35	0.93	1.13	*
9	Sinohara_4_I_PH	herbivory	pollination	30	22	0.77	0.54	0.67	*
10	Melian_OO_OO_PH	pollination	herbivory	208	148	2.08	1.12	0.75	*
11	Hackett_1_ALL_PH	pollination	herbivory	263	81	1.54	1.42	0.98	*
12	Hackett_2_ALL_PH	pollination	herbivory	74	44	0.92	0.83	0.91	*
13	Hackett_1_S_PH	pollination	herbivory	85	26	0.85	0.81	0.88	
14	Hackett_1_GL_PH	pollination	herbivory	106	30	1.10	1.08	0.58	*
15	Pocock_OO_OO_PH	pollination	herbivory	308	142	1.31	1.63	0.62	
16	Melian_OO_OO_HSD	herbivory	dispersion	148	81	1.24	0.75	1.30	
17	McFayden_ALL_A_HPa	herbivory	parasitism	389	265	1.29	0.88	1.39	
18	McFayden_1_A_HPa	herbivory	parasitism	104	76	1.16	0.48	1.24	
19	McFayden_2_A_HPa	herbivory	parasitism	92	66	1.21	0.63	1.35	
20	McFayden_3_A_HPa	herbivory	parasitism	116	63	0.98	0.51	1.10	
21	McFayden_4_A_HPa	herbivory	parasitism	104	71	1.01	0.45	0.98	*
22	McFayden_5_A_HPa	herbivory	parasitism	121	71	1.18	0.55	1.17	*
23	McFayden_6_A_HPa	herbivory	parasitism	94	53	1.12	0.50	1.12	
24	McFayden_7_A_HPa	herbivory	parasitism	106	52	1.04	0.44	0.93	*
25	McFayden_8_A_HPa	herbivory	parasitism	109	85	1.33	0.60	1.38	
26	McFayden_9_A_HPa	herbivory	parasitism	79	45	1.05	0.31	1.31	
27	McFayden_10_A_HPa	herbivory	parasitism	113	64	1.23	0.47	1.32	
28	McFayden_ALL_B_HPa	herbivory	parasitism	335	225	1.28	0.87	1.20	*
29	McFayden_1_B_HPa	herbivory	parasitism	117	75	0.99	0.47	1.07	
30	McFayden_2_B_HPa	herbivory	parasitism	78	45	1.13	0.51	0.89	*
31	McFayden_3_B_HPa	herbivory	parasitism	105	60	0.99	0.53	0.96	*
32	McFayden_4_B_HPa	herbivory	parasitism	84	43	1.16	0.54	1.05	*
33	McFayden_5_B_HPa	herbivory	parasitism	73	44	1.34	0.36	1.37	
34	McFayden_6_B_HPa	herbivory	parasitism	86	59	0.84	0.33	0.89	
35	McFayden_7_B_HPa	herbivory	parasitism	83	66	0.92	0.31	1.11	
36	McFayden_8_B_HPa	herbivory	parasitism	82	56	1.12	0.53	1.08	*
37	McFayden_9_B_HPa	herbivory	parasitism	68	41	0.80	0.51	0.85	
38	McFayden_10_B_HPa	herbivory	parasitism	84	45	0.97	0.34	1.07	
39	Hackett_1_ALL_HPa	herbivory	parasitism	54	31	0.64	0.16	0.73	
40	Hackett_1_WL_HPa	herbivory	parasitism	28	14	0.42	0.21	0.33	*
41	Melian_OO_OO_PSD	pollination	dispersion	208	81	1.66	1.12	1.30	*
42	Dattilo_OO_OO_PSD	pollination	dispersion	266	73	1.14	1.11	1.27	
43	Dattilo_OO_OO_PA	pollination	ant	267	70	1.09	1.12	0.79	

Table B: Degree heterogeneity of the shared set species in the tripartite networks in the data-set. Different columns show the name of the layer, the number of species in the layer (N), the degree heterogeneity of all shared set species in the tripartite network (LS_HD), and also for the subset present in each of the two bipartite networks that compose the tripartite. Networks marked with a star indicates those where the degree heterogeneity of the shared set species in the merged (i.e. tripartite) network is larger than that of the subset of shared set species included in each of the two bipartite networks composing the tripartite.

3 Interdependence

We study how the Robustness of the two animal species sets are correlated in the tripartite networks of our database when plants were randomly driven to extinction. A positive correlation means that the same plant species that are important for the survival of the two sets of animal species (i.e. plants important for pollinators are also important for herbivores). However, if the relevant plants are different, deleting a given plant will cause a large loss of animal species in one set, but not have much effect in the other set, rendering no interdependence.

We find that, in general, the correlation between the Robustness, henceforth called interdependence (I) was either positive or null in all the networks. However, some MA and MM networks ($\sim$20% of all networks in the database) show a negative correlation (albeit very weak in most cases). The value of I found in AA networks (Fig EA) is on average significantly higher from that found in MM and MA networks, mirroring previous results where the correlations between species sets that where linked by feeding interactions were found to be stronger [7].

Comparison with the four null models indicates that, for AA networks, the positive interdependence may be a result of the process in which secondary extinctions take place (i.e. cascading extinctions), since the empirical value is not significantly different from the null expectation in most cases (see Fig E B to E). As for MM and MA networks, comparison with all null models shows that the structure of empirical networks (in particular the degree heterogeneity of the species, Fig E B and C) makes the two layers more decoupled than expected by chance, since the null models predict in fact a negative Interdependence. In the two null models where degree heterogeneity is conserved the empirical interdependence is not different from the null expectation in any case (Fig E D and E).

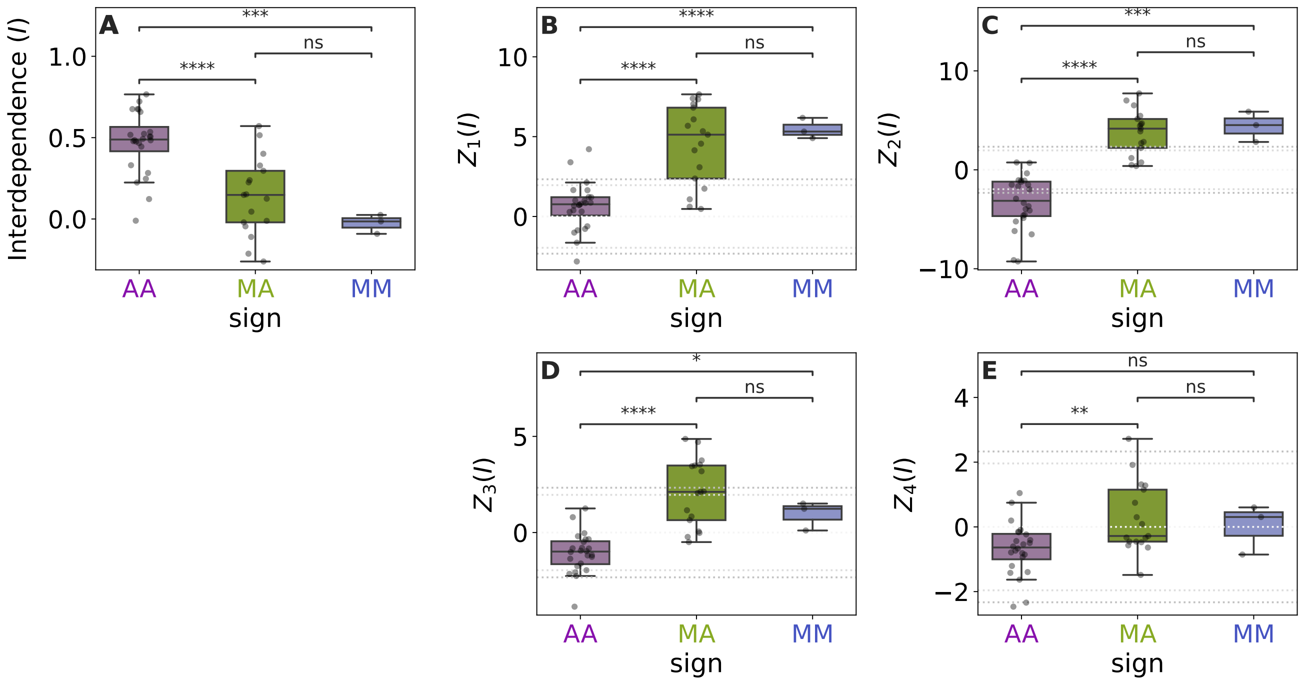

Fig E: A: Interdependence (I) in the tripartite networks in our data set when plants are randomly driven to extinction, grouped and color coded by the signs of their interaction layers. B to E: Z-score of interdependence (I) in the different null models. The horizontal lines represent the Z-score values associated with a 95% and 98% confidence interval, z=1.96 and z=2.33 respectively.

Effect of structural features on interdependence

The different ways in which the two interaction layers are connected is reflected also in how the structural features of the connector nodes are able to explain the interdependence. In MM and MA networks, these structural features play an important role: H_C and PR_c alone account for $\sim$55% and $\sim$20% of the variance in interdependence, respectively (see Fig FE and F). In AA networks, in contrast, the structural metric that best explains the variance in interdependence is C, but only $\sim$16% of it (see Fig FD). In MM and MA networks, a multiple linear regression explains 64% of the interdependence as a function of H_C (how frequently hubs are connector nodes) and PR_c (the average participation ratio of the connector nodes) and degree heterogeneity, as shown in Table C. No structural metric explains more than 16% of the variance in interdependence in AA networks.

Regarding the presence of (weak) anti-correlations found in MA and MA networks, one could think that their origin lies in the fact that not all the targeted plant species are shared between the two layers in MA and MM networks. In those cases, secondary extinctions can happen in only one of the two interaction layers, while the other remains intact, introducing a

Table C: Multiple linear regression of interdependence (I) vs structural features

	I			
	AA(all)	MA & MM(all)	AA(best)	MA & MM(best)
$\sigma_k / < k >$	-0.03	0.31		0.30
	(0.29)	(0.19)		(0.18)
C	0.52**	-0.03	0.40*	
	(0.22)	(0.15)	(0.20)	
H_C	-0.29	0.71***		0.70***
	(0.24)	(0.15)		(0.14)
PR_C	-0.09	0.51**		0.50**
	(0.28)	(0.19)		(0.18)
AIC	71.89	42.67	67.92	40.72
Observations	24	20	24	20
R^2	0.23	0.70	0.16	0.70
Adjusted R^2	0.07	0.62	0.12	0.64
Residual Std. Error	0.99	0.63	0.96	0.61
F Statistic	1.41	8.75***	4.20*	12.41***

Note: *p<0.1; **p<0.05; ***p<0.01

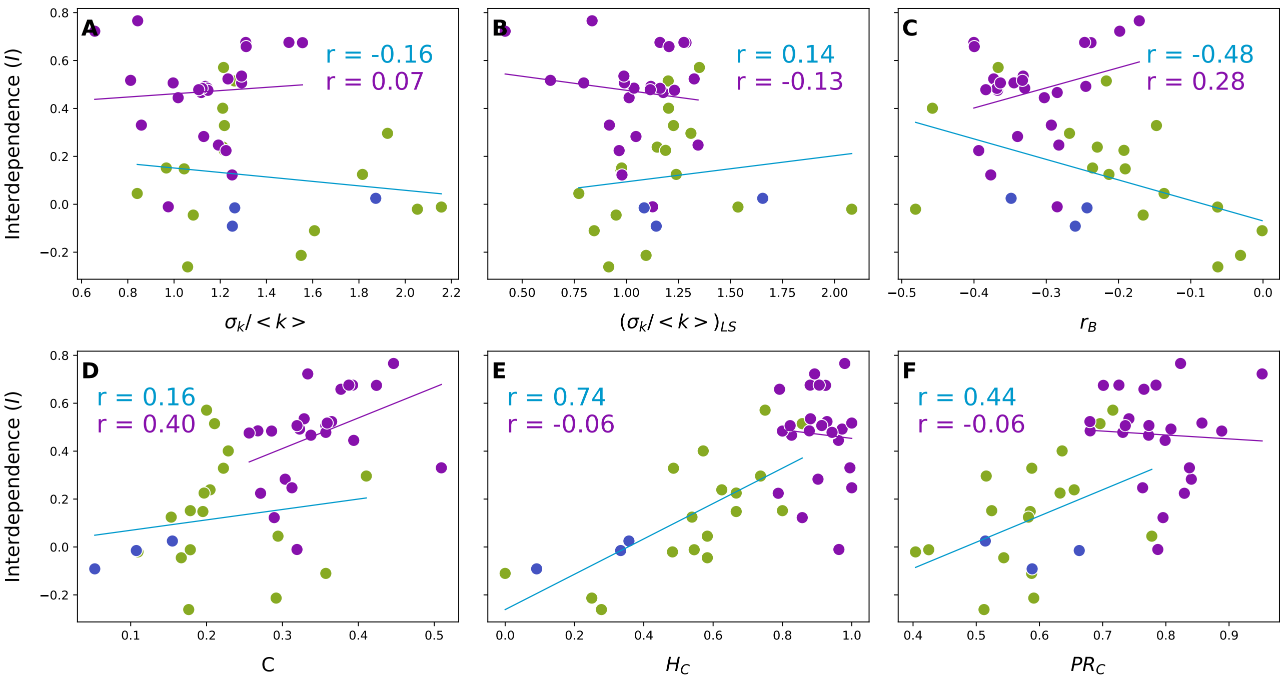

Fig F: Correlation of interdependence (I) vs the structural features studied: A) degree heterogeneity ($\sigma_k / < k >$), B) degree heterogeneity of the shared set species ($\sigma_k / < k >_{LS}$), C) degree-degree correlations (r), D) proportion of connector nodes inside the shared set (C), E) Proportion of shared set hubs that are connectors (H_C), and F) average participation ratio of the connector nodes (PR_C) on the tripartite networks in our data set. The colors of the points represent the different types of tripartite networks according to the sign of their interaction layers (see legend). The values in the lower right side are the Pearson correlation coefficients considering only MM and MA networks (blue), only AA networks (violet).

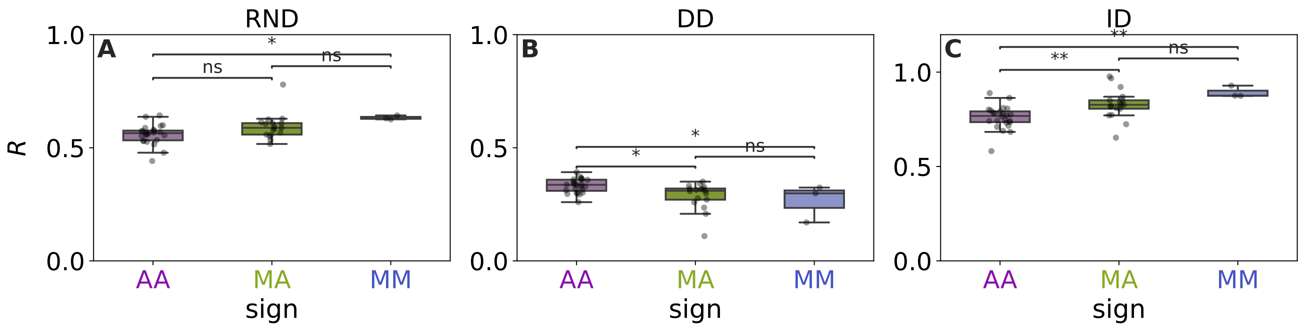

Fig G: Robustness of the tripartite networks in our data set in three different extinction scenarios: A) random plant extinction (RND), B) extinction of plants by decreasing degree (DD), i.e. generalist plants are attacked first, and C) extinction of plants by increasing degree (ID), i.e. specialist plants are attacked first.

trivial anti-correlation between the Robustness of the two interaction layers. However, the panel D of Fig F shows that there is a very low correlation between the proportion of shared species in the shared set (C) and the interdependence I in MA and MM networks (Pearson $r \approx 0.16$). In fact, we find that the quantity that best correlates with I in MM and MA networks ($r \approx 0.75$) is the tendency of the 20% of the most connected nodes in the shared set to be connector nodes (FigFE). That is, the fact that the generalist species in the shared set tend to be involved in the two interaction layers or not makes the difference in terms of interdependence in MA and MM networks. If the generalist plants tend to be involved in both interactions, then the interdependence is larger. If, on the contrary, the generalist plants are involved in only one of the two interaction layers, then we see no correlation, as in MM networks, or even an anti-correlation. In AA networks, on the contrary, the structural metric that best correlates with I (albeit weakly) is the proportion of connector nodes in the shared set (C). Since, as said before, for a parasitoid to undergo extinction the herbivore host has to go extinct first, it is not a surprise that the higher the number of connectors, the higher the interdependence of the two interaction layers.

4 Robustness

All types of tripartite networks seem to react in a similar way (with Robustness in the same range of values) to the three extinction scenarios explored, although some differences between network types were significant (see Fig GA to C). In line with previous studies on networks with only one interaction type, all ecological tripartite networks were most fragile when plants were selectively attacked targeting the most connected plants first (DD scenario), and the least fragile when plants were attacked selecting the specialists plants first (ID scenario), as previously reported in mutualistic plant-pollinator, food-webs and host-parasite networks (albeit fish-parasites, not herbivore-parasites) [14, 10, 15, 16, 17]. In the random extinction scenario (RND scenario), Robustness showed intermediate values, with AA networks being the more fragile of the three different type of networks and MM networks the more robust (see Fig GA).

Effect of basic structural features in Robustness

Table D: Multiple regression of Robustness (R) vs structural features

	R			
	AA(all)	MA & MM(all)	AA(best)	MA & MM(best)
$\sigma_k / < k >$	0.71***	0.57**	0.68***	0.38*
	(0.16)	(0.26)	(0.14)	(0.19)
C	0.21	-0.59**	0.24**	-0.51**
	(0.12)	(0.20)	(0.11)	(0.19)
H_C	0.08	0.22		
	(0.13)	(0.20)		
PR_C	-0.22	0.23	-0.25*	
	(0.15)	(0.26)	(0.14)	
AIC	43.2	54.57	41.61	53.24
Observations	24	20	24	20
R^2	0.77	0.46	0.76	0.38
Adjusted R^2	0.72	0.31	0.73	0.31
Residual Std. Error	0.54	0.85	0.53	0.86
F Statistic	15.59***	3.15**	21.39***	5.18**
Note:			*p<0.1; **p<0.05; ***p<0.01	

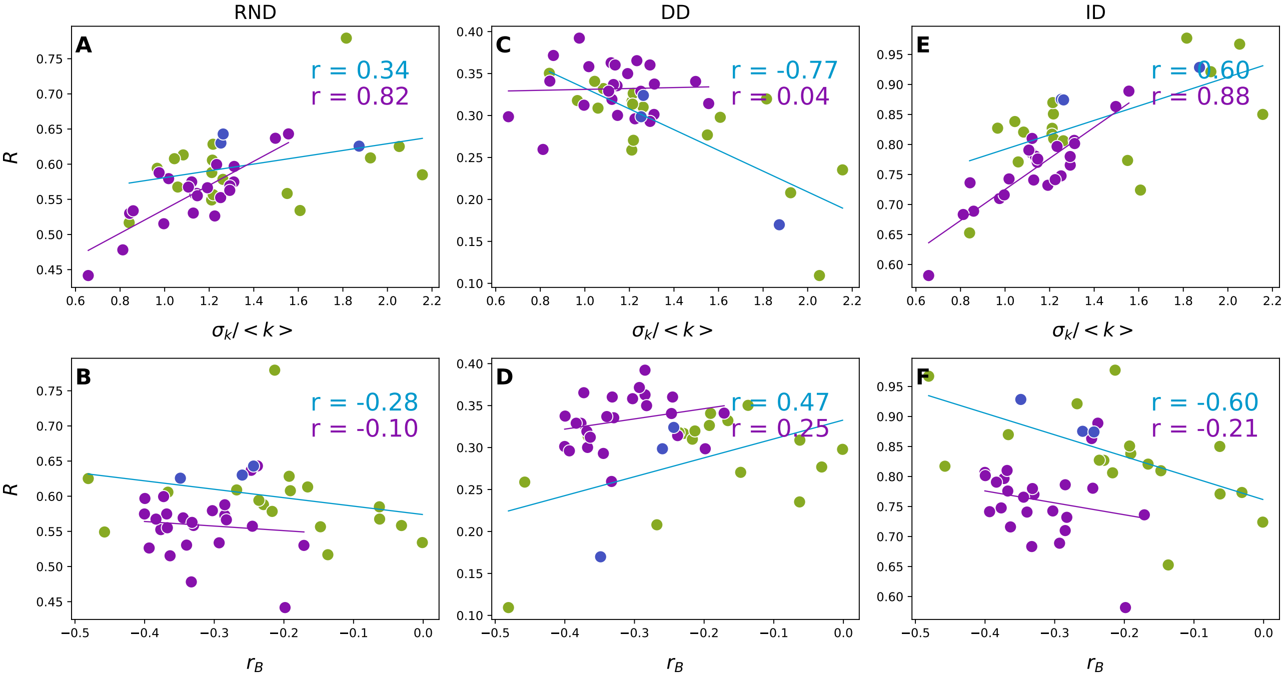

Fig H: Effect of degree heterogeneity ($\sigma_k/ < k >$) and degree-degree correlations (r) on the Robustness (R) of the tripartite networks in our data set. The colors of the point represent the different types of tripartite networks according to the sign of their interaction layers (violet AA, green MA, and blue MM). The values in the upper right side are the Pearson correlation coefficients considering only MM and MA networks (blue), and only AA networks (violet). The lines represents the best linear regression for each of the correlations.

Studying how the basic structural features of the tripartite networks affect their robustness to plant loss, we find that the effects of the different structural features greatly depend on the explored extinction scenario (see Fig H).

We did a multiple linear regression of the Robustness (R) in the RND extinction scenario as a function of the different structural features (Table D)

Degree heterogeneity is associated with an increase in the Robustness of all tripartite networks in the RND and ID scenarios ($r_{RND} = -0.62$, $r_{ID} = -0.75$), but increasing it in MA and MM networks in the DD scenario ($r_{DD} = 0.73$). The positive effect of degree heterogeneity on robustness was already reported for bipartite mutualistic networks in [18] (through nestedness, but it was also shown that nestedness is consequence of degree heterogeneity [13]). These results are also in line with previous studies showing that ecological networks are quite robust to the extinction of the most specialist species, but quite fragile if the most generalized species were the ones going extinct [11, 14, 10]. This robustness to random extinctions has largely been explained by their heterogeneous structure, in food webs [11] and in plant-mutualistic networks [10], and is also present in the tripartite networks in our data set.

Relevance of the empirical structure

To gauge the relevance of the empirical structure in determining Robustness in the RND extinction scenario, we tested how the Robustness of the empirical networks compared to that of their different randomizations. AA empirical networks where consistently more robust than their randomizations when the original degree distribution was lost (i.e., in all null models except “4”, see Fig JB to D). Empirical MA and MM networks, on the other hand, were less robust than their randomizations in ensemble “1” (and MM networks also in ensemble “2”). This indicates two things. First, that the degree distribution is a mayor driver of Robustness, since as long as it is kept constant (null model “4”) the Robustness of all types of empirical networks is not significantly different from the random expectation. Second, that the degree distribution of empirical AA networks is such that makes them more robust to the loss of plant species.

We also compared the robustness of the MA and MM tripartite networks with that of the two bipartite networks that compose the tripartite to see if considering multiple interactions at the same time enhanced or hindered the robustness of the whole community. This analysis was only possible in MA and MM networks, since in AA networks plants appear only in one of the two bipartite networks –plant-herbivores– making it impossible to quantify Robustness in the other bipartite network –herbivore-parasite–We find that, in all cases, considering the merged network did not increased the robustness to plant extinction, a result in line with previous findings in multiple-mutualistic ecological networks [8]. Rather, the robustness of the whole community (R) was in between that of the two bipartite networks composing the tripartite network (Fig KD).

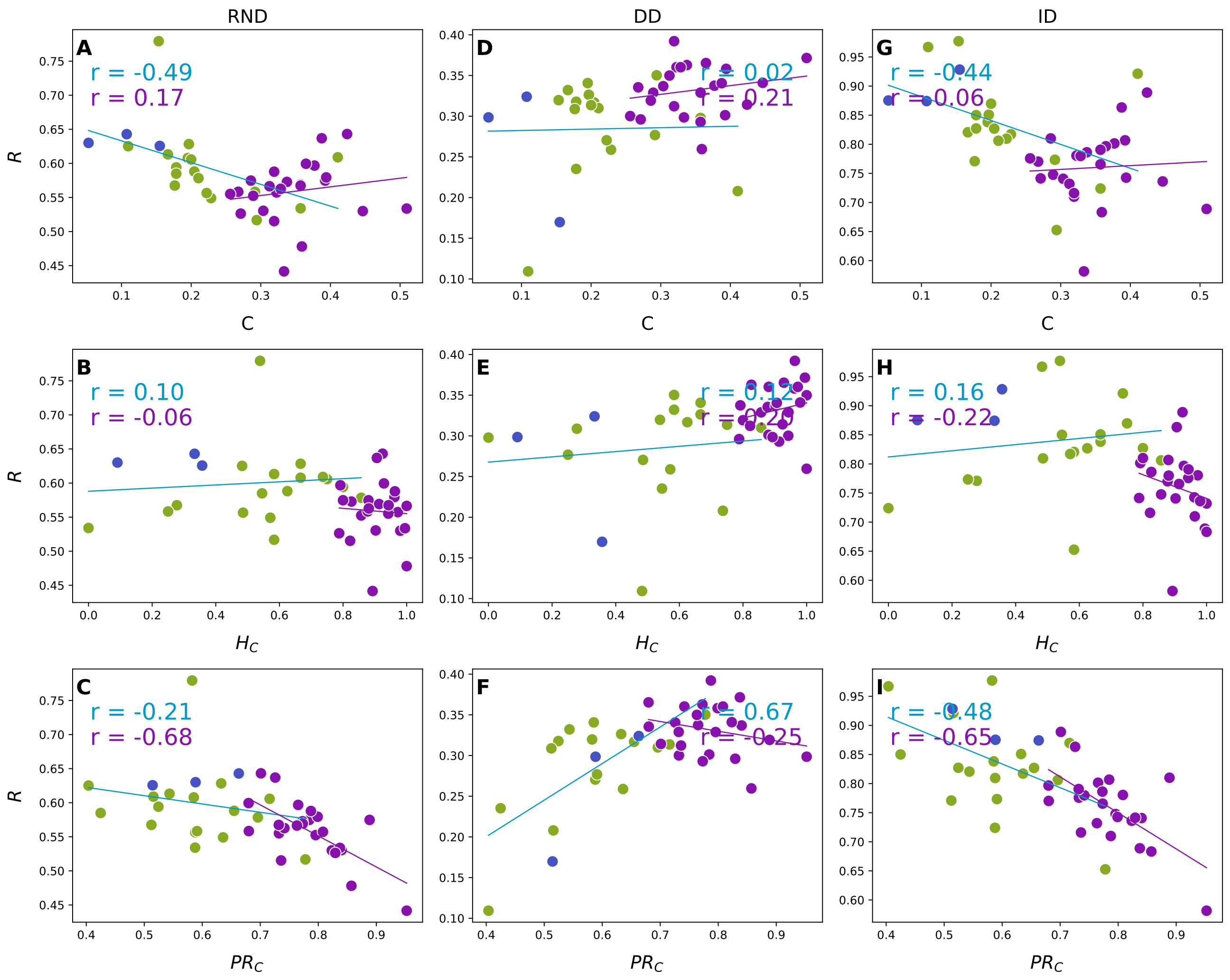

Fig I: Effect of structural features of the shared set on the Robustness (R) of the tripartite networks in our data set. The color of the points represents the different types of tripartite networks according to the sign of their interaction layers (violet AA, green MA, and blue MM). The values on the side are the Pearson correlation coefficients considering only MM and MA networks (blue), and only AA networks (violet). The lines represent the best linear regression for each of the correlations.

We investigate whether one of the two bipartite networks could be driving the values of the Robustness of the tripartite network (R), and we find that the larger (i.e. species richer) bipartite network could account for about 80% of the variance in the Robustness of the whole community (Fig KA) while the Robustness of the smaller (i.e. species poorer) bipartite network could account for only 46% of the variation (Fig KB). Interestingly, the robustness of the whole community could be expressed as a linear combination of the robustness of the larger and smaller bipartite networks composing the tripartite network (Fig KD), with the larger one having a larger contribution to the Robustness of the tripartite network (0.74) and the smaller one a smaller contribution (0.25). As we see in Fig KC, there is a very good agreement between the measured values of Robustness in the empirical tripartite networks (R) and the Robustness estimated from the two Robustness of the independent bipartite networks ($R_{(est)}$), highlighting the importance of considering the multiple interactions to better estimate the robustness of the whole community.

Using more advances extinction algorithms

Through the main text, we employ the classical co-extinction algorithm [10] to measure robustness to plant losses. Although this approach lacks realism (since there are no ecological dynamics behind it, nor any information about species preferences), it has proven useful in understanding the threat that biodiversity loss poses to ecosystem services and functioning [12, 10, 7]. To verify that the classical approach provides a lower bound on the damage caused on the ecological community (i.e. is the most conservative approach), we implemented the stochastic version of the co-extinction algorithm in mutualistic-antagonistic networks[19]. In this stochastic version, a species i will undergo a secondary extinction following the extinction of plant j with a probability $P_{ij} = R_i.d_{ij}$, where d_{ij} is the dependency of species i on j (interaction weight), and R_i represents the intrinsic demographic dependence of species i on mutualism (for example, it may reflect the intrinsic dependence of

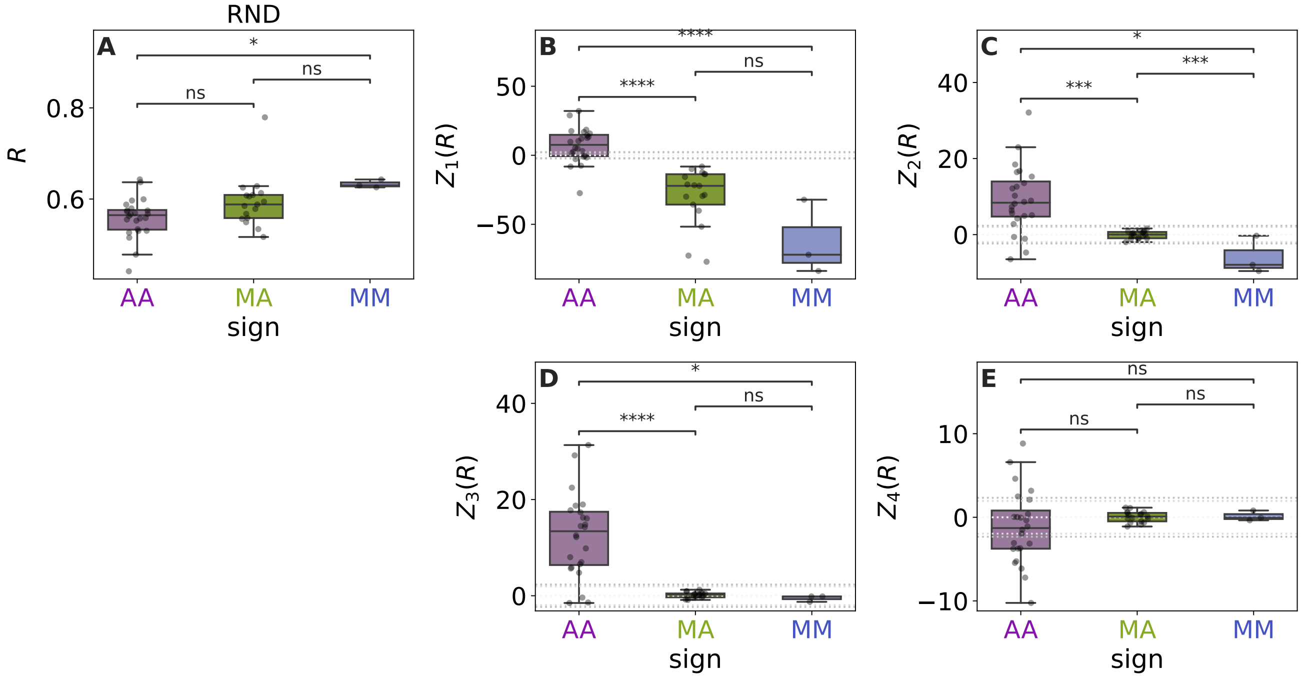

Fig J: A: Robustness (R) of the tripartite networks in our data set when plants are randomly driven to extinction, grouped and color coded by the sign of the interaction layers. B to E: Z-score of R in the different null models. The horizontal lines represent the Z-score values associated with a 95% and 98% confidence interval, z=1.96 and z=2.33 respectively.

a pollinator on nectar or on other resources, so that a lower value of R_i would mean that that animal species does not rely completely on mutualism to survive, or that a plant can also rely on self pollination to survive). We simulated extinction cascades using this algorithm in the following manner: We erased plants following the extinction sequence. At each plant extinction, their animal partners had a probability P_ij of undergo extinction. In our case, to simulate obligate mutualism we fixed $R_i = 1$ for all animal species, but we continued using a bottom up control scenario by fixing R_0 for plants (i.e. plants could only go extinct in their turn on the extinction sequence, even if they had no mutualistic partners).

Below we show the comparison between our results in the main text (using the classical approach) and those obtained with the stochastic model on weighted networks, when available. When weights were not provided, we considered all equal weights of 1, which stills allows to implement the stochastic version of the co-extinction model.Fig L shows that the values of robustness (left) and Interdependence (right) are similar independently of the algorithm used, in particular for interdependence they are almost equivalent. Note that in the left figure, the values in the horizontal axis (robustness) are always higher than their stochastic weighted counterparts in the vertical axis, which corroborates that the classical approach at quantifying robustness is the most conservative, providing a clear lower bound that does not depend on any further parameter.

Fig M shows that the result of robustness of the merged network being a composition of the robustness of larger and smallest interaction layers also holds also in the case of using the stochastic weighted version.

In light of these results, the classical approach provides a lower bound on the damage caused to the ecological community. Given the fact that is also a more parsimonious model, without any parameter, we decided to use the classical approach in the main text.

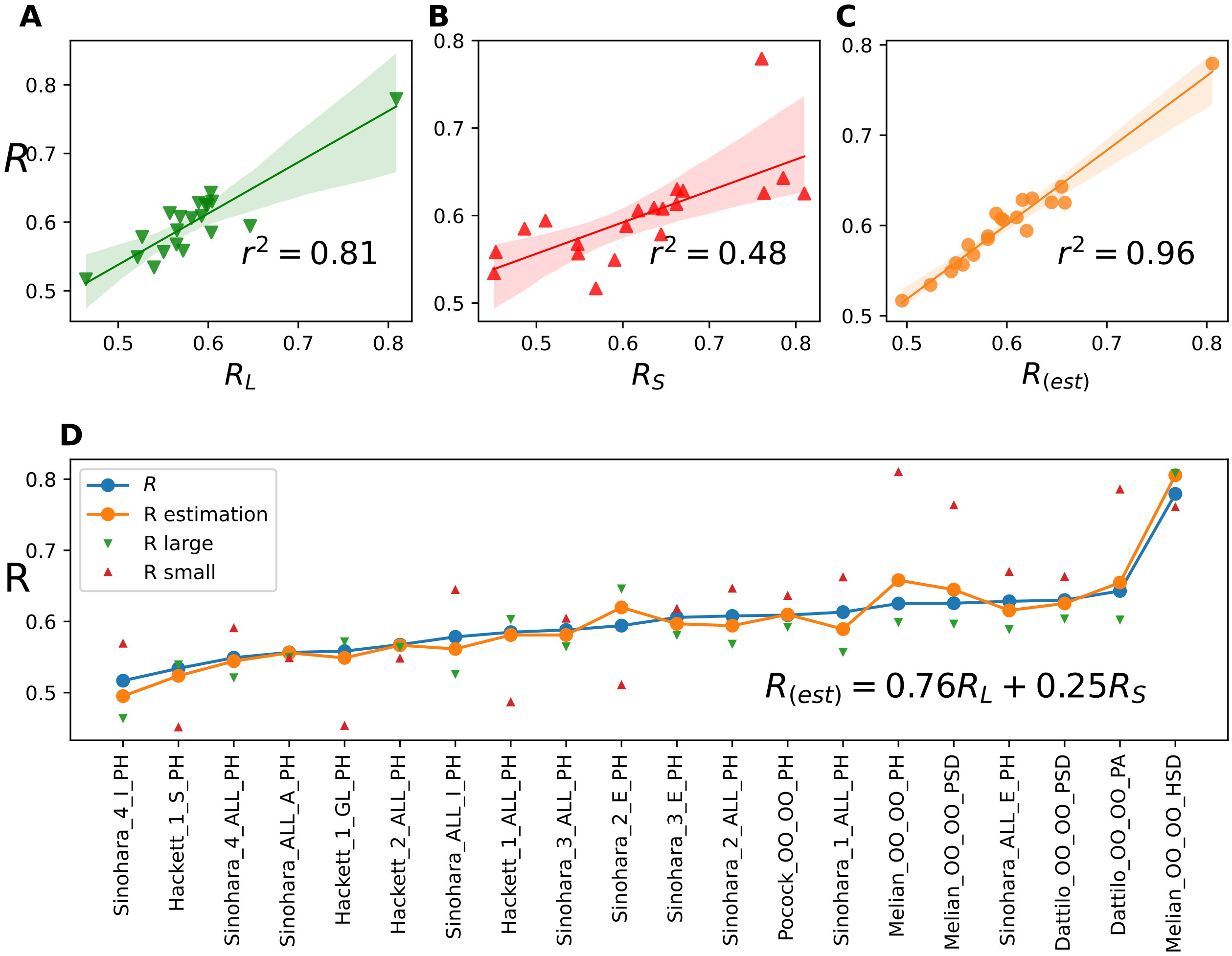

Fig K: Upper panel: correlation of the Robustness of the whole merged network (R) with A) the Robustness of the larger interaction layer (R_L), B) the Robustness of the smaller interaction layer (R_S), and C) with the best estimation of Robustness ($R_{(est)}$) as a composition of the Robustness of the two interaction layers. Lower panel: Comparison of the Robustness of the tripartite networks (in blue) with that of the two interaction layers composing them (R_L in green and R_S in red) and with the best estimated Robustness as a composition of the Robustness of the two interaction layers (formula shown).

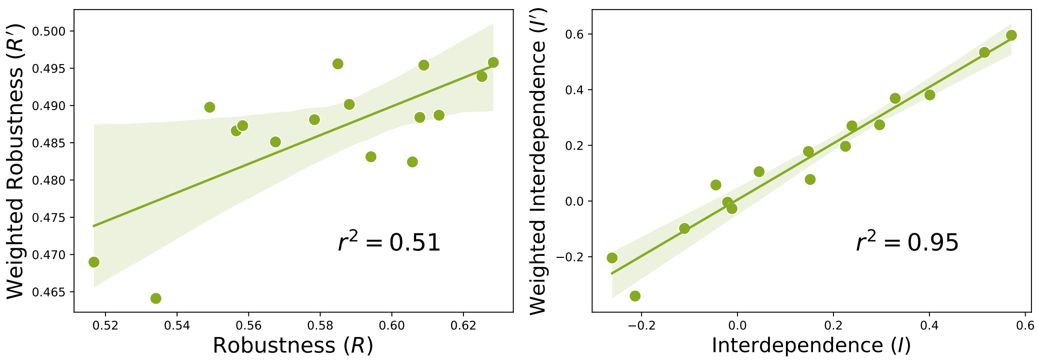

Fig L: Comparative of results between the stochastic co-extinction algorithm in weighted networks (when available) with the results of the classic co-extinction algorithm used in the main text, for Robustness (left) and Interdependence (right).

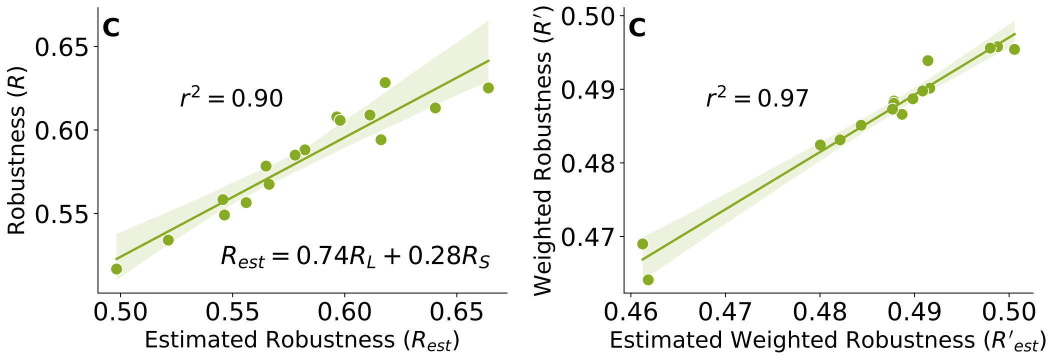

Fig M: Comparative of the estimated robustness of the mutualistic-antagonistic tripartite networks as a combination of the robustness of the larger and smaller bipartite networks using the basic algorithm (left, as in the main text) and using the stochastic version of the algorithm and weighted networks (right).

5 Plant importance

The histogram of importance of plants indicates that the ranking is particularly sensitive in the case of the more important plants, both in the particular case that we use as an example in the main text (Fig N) as when we consider all the communities together (Fig O). While it is more difficult to differentiate between the less important plants (those with lower values of importance), the ranking is well defined, as can be seen from the correlation values between the importance and the ranking based on importance (lower row of Fig. R.N). Also, the whole community ranking is more spread than any of the two other rankings alone, indicating that considering both interactions simultaneously can help to better gauge the importance of plants.

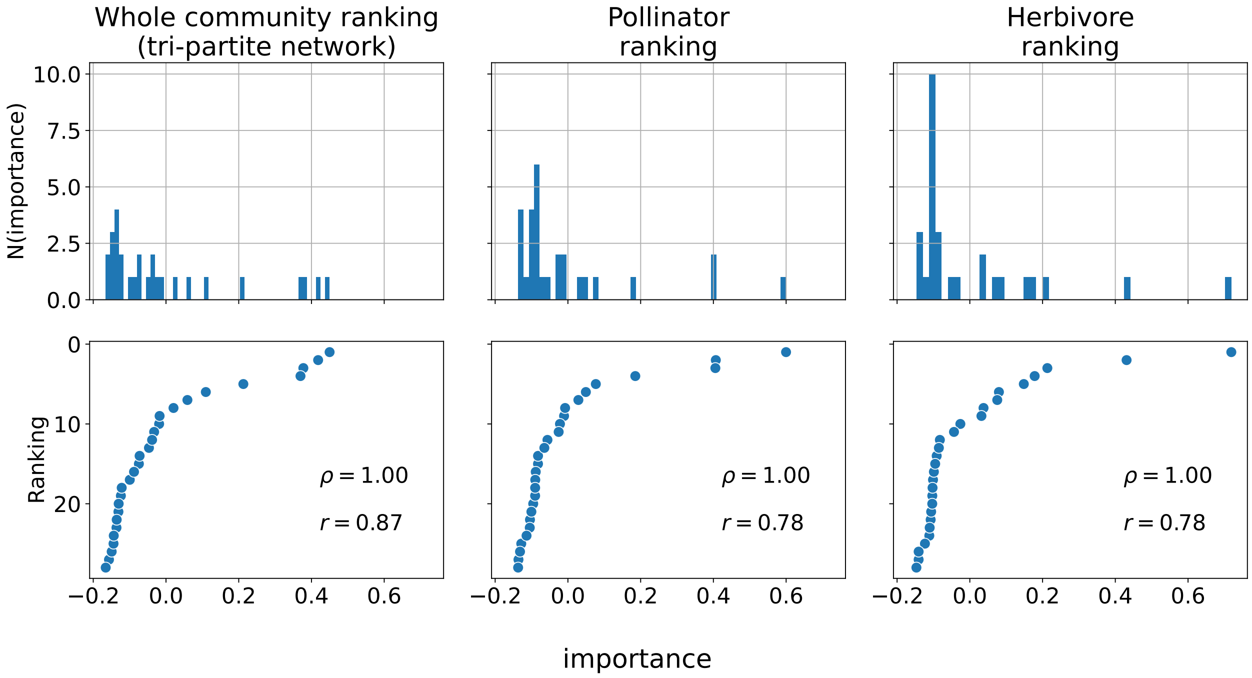

Fig N: Upper row: Distribution of plant importance values in the network used as example in Fig. 4. The histograms depict the amount of plants with a given importance in the whole community (left), with a given importance for pollinators (center) and for herbivores (rigth). Lower row: Ranking of importance (based on importance) vs importance. Inset values show the spearman rank correlation coefficient (ρ) and pearson correlation coefficient (r).

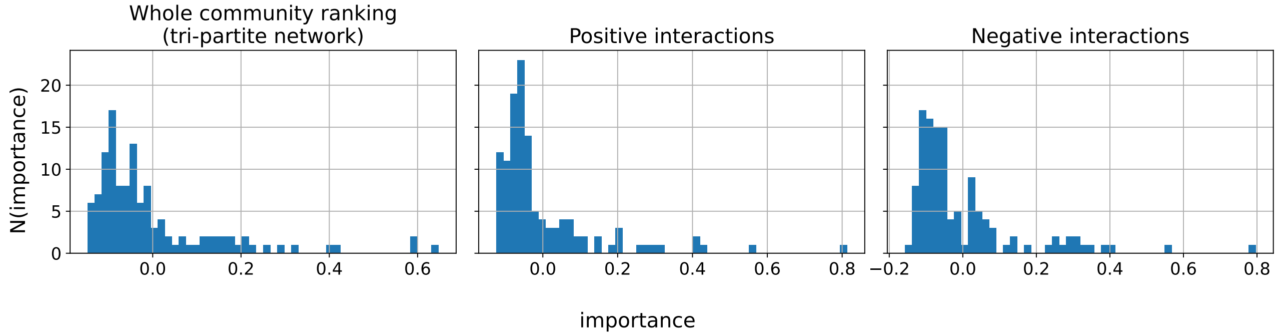

Fig O: Distribution of plant importance values considering all the communities together. The histograms depict the amount of plants with a given importance in the whole community (left), with a given importance for animals connected through positive interactions -pollination or seed dispersion- (center) and for animals connected through negative interactions -herbivory- (rigth).

6 Connector nodes

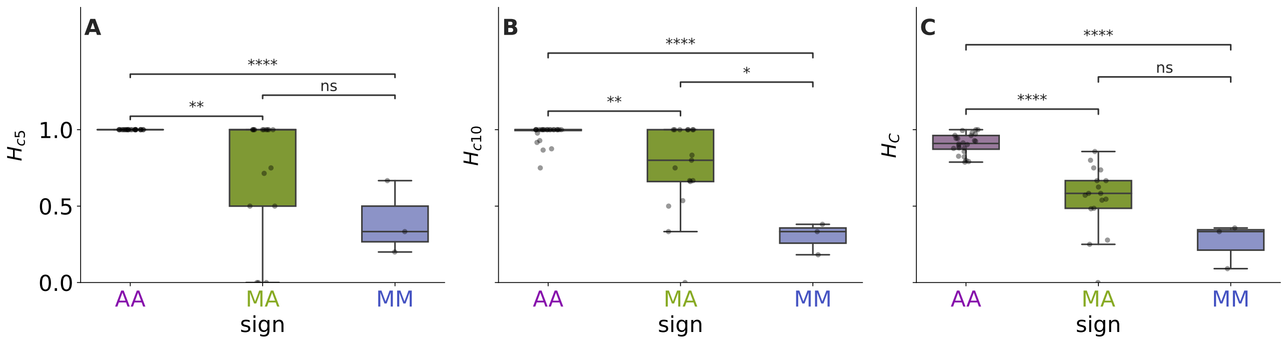

Fig P: Effect of the threshold considered in determining the proportion of shared set hubs that are connector nodes (H_C), i.e. the proportion of connector nodes inside the 5% (A), 10% (B) or 20% (C) of most connected shared set nodes.

7 Null models and Z-scores

To assess the importance of network structure in determining a certain network feature, we compared measurements of that feature performed on empirical networks with measurements performed on randomized versions of those networks keeping some properties fixed. We use four different null-models (see Fig 5 in main text), going from the least restrictive to the most restrictive (left to right in the figure), they are:

- "1", or “NL”: the number of nodes in each of the three species sets (N_a, N_b, and N_l), as well as the number of links in each interaction layer (L_α, L_β) are kept constant. The randomization is performed in both interaction layers (α and β) separately, by linking $L_\alpha(L_\beta)$ pairs of nodes from the two different sets involved in the interaction: a and l (b and l), ensuring that each of the nodes in the original network receives at least one link. Networks generated in the “constant NL” ensemble have the same size and connectance as the empirical networks, but erase any other structure present in the original interactions. In particular, the degree distribution of each species set is lost as well as the degree-degree correlations between nodes of species sets. When a metric is similar in the empirical networks and in the constant NL ensemble, one knows that the number of species and the connectance are the relevant features determining that metric.
- "2", or “NL2”: the number of nodes in each species set (N_a,N_b, N_l) is kept constant, as well as the number of interactions in each interaction layer (L_α,L_β), and the degree of all nodes except those in the shared set. This null model allows the study of the effect of the degree distribution of the shared set species has in a given metric. When a metric is similar in the empirical network and in this random ensemble, one knows that the size of the sets and connectance are the the relevant features determining that metric (i.e. the degree distribution of the shared set species does not play a relevant role).
- "3", or “K2”: the number of nodes in each species set (N_a,N_b, N_l) is kept constant, as well as the degree distribution of the species in both interaction layers. However, the nodes in the shared set do not keep their total degree (i.e. we beak the correlation between the degree of the shared set nodes in each interaction layer). This null model allows the study of the effect the correlation between the degree of connector nodes in each interaction layer has in a given metric. When a metric is similar in the empirical network and in this ensemble one knows that the correlation in the degrees of the connector species in different interaction layers is not relevant in determining that given metric.
- "4", or “K”: the number of nodes in each species set (N_a,N_b, N_l), the total number of links in each interaction layer (L_α,L_β), and each node’s degree (the number of links of a given node) are kept constant. However, the identity of neighbours are reshuffled within a layer. The reshuffling of the empirical networks is performed using the ‘curveball algorithm’, that ensures an unbiased sampling of random matrices [20]. Networks generated in the “constant K” ensemble have exactly the same degree distribution as the empirical networks, but erase any other structure present in the original interactions, in particular the degree-degree correlation. When a metric is significantly different in the empirical network and in this null model, one knows that it is due to other structural features than degree distribution. When a metric is similar in the empirical networks and in the constant K ensemble, one knows that size and degree distribution are the relevant features determining that metric.

To compare the value of a given metric in an empirical network with that obtained in the random ensemble, we measure the Z-Score of that metric. The Z-Score quantifies the number of standard deviations by which the value of a raw score (i.e., the value measured in the empirical network) is above or below the mean value of what is being measured in the random ensemble, and is defined as:

$$Z = \frac{x - \bar{x}}{\sigma_x} \tag{1}$$

where $\bar{x}$ is the average x measured in the random ensemble and σ_x its standard deviation. Positive/negative values mean that the measure in the empirical network is higher/lower than in the random-ensemble. The relevant Z score values when using a 95% confidence level are -1.96 and +1.96. The p-value associated with a 95% confidence level is 0.05. If the Z-Score is between -1.96 and +1.96, the p-value will be larger than 0.05, and one cannot reject the null hypothesis; Conversely, for a measure to be outside the 98% confidence interval (p<0.02) the absolute value of the Z-score has to go up to 2.33.

In the case where one wants to compare two distributions, for example when comparing the Robustness in the empirical network and the random ensemble (note that the Robustness of the empirical networks is a distribution of values rather than a number) we use the z-test. In this case, we compute the Z-score as:

$$Z = \frac{(\bar{x_1} - \bar{x_2})}{\sqrt{\sigma_{x_1}^2 + \sigma_{x_2}^2}} \tag{2}$$

where $\bar{x_1}$ is the mean value of sample one (empirical network), $\bar{x_2}$ is the mean value of sample two (randomized ensemble), σ_{x_1} is the standard deviation of sample one divided by the square root of the number of data points, and σ_{x_2} is the standard deviation of sample two divided by the square root of the number of data points. .

References

[1] Macfadyen S, Gibson R, Polaszek A, Morris RJ, Craze PG, Planqué R, et al. Do differences in food web structure between organic and conventional farms affect the ecosystem service of pest control? Ecology Letters. 2009;12(3):229–238. doi:10.1111/j.1461-0248.2008.01279.x.

[2] Massicotte P, South A. rnaturalearth: World Map Data from Natural Earth; 2023. Available from: https://CRAN.R-project.org/package=rnaturalearth.

[3] R Core Team. R: A Language and Environment for Statistical Computing; 2023. Available from: https://www.R-project.org/.

[4] Shinohara N, Uchida K, Yoshida T. Contrasting effects of land-use changes on herbivory and pollination networks. Ecology and Evolution. 2019;9(23):13585–13595. doi:10.1002/ece3.5814.

[5] Melián CJ, Bascompte J, Jordano P, Krivan V. Diversity in a complex ecological network with two interaction types. Oikos. 2009;118(1):122–130. doi:10.1111/j.1600-0706.2008.16751.x.

[6] Hackett TD, Sauve AMC, Davies N, Montoya D, Tylianakis JM, Memmott J. Reshaping our understanding of species' roles in landscape-scale networks. Ecology Letters. 2019;22(9):1367–1377. doi:10.1111/ele.13292.

[7] Pocock MJO, Evans DM, Memmott J. The Robustness and Restoration of a Network of Ecological Networks. Science. 2012;335(6071):973–977. doi:10.1126/science.1214915.

[8] Dáttilo W, Lara-Rodríguez N, Jordano P, Guimarães PR, Thompson JN, Marquis RJ, et al. Unravelling Darwin's entangled bank: architecture and robustness of mutualistic networks with multiple interaction types. Proceedings of the Royal Society B: Biological Sciences. 2016;283(1843):20161564. doi:10.1098/rspb.2016.1564.

[9] Jordano P, Bascompte J, Olesen JM. Invariant properties in coevolutionary networks of plant-animal interactions. Ecology Letters. 2002;6(1):69–81. doi:10.1046/j.1461-0248.2003.00403.x.

[10] Memmott J, Waser NM, Price MV. Tolerance of pollination networks to species extinctions. Proceedings of the Royal Society of London Series B: Biological Sciences. 2004;271(1557):2605–2611. doi:10.1098/rspb.2004.2909.

[11] Solé RV, Montoya M. Complexity and fragility in ecological networks. Proceedings of the Royal Society of London Series B: Biological Sciences. 2001;268(1480):2039–2045. doi:10.1098/rspb.2001.1767.

[12] Dunne JA, Williams RJ, Martinez ND. Food-web structure and network theory: The role of connectance and size. Proceedings of the National Academy of Sciences. 2002;99(20):12917–12922. doi:10.1073/pnas.192407699.

[13] Jonhson S, Domínguez-García V, Muñoz MA. Factors Determining Nestedness in Complex Networks. PLoS ONE. 2013;8(9):e74025. doi:10.1371/journal.pone.0074025.

[14] Dunne JA, Williams RJ, Martinez ND. Network structure and biodiversity loss in food webs: robustness increases with connectance. Ecology Letters. 2002;5(4):558–567. doi:10.1046/j.1461-0248.2002.00354.x.

[15] Bascompte J, Stouffer DB. The assembly and disassembly of ecological networks. Philosophical Transactions of the Royal Society B: Biological Sciences. 2009;364(1524):1781–1787. doi:10.1098/rstb.2008.0226.

[16] Dallas T, Cornelius E. Co-extinction in a host-parasite network: identifying key hosts for network stability. Scientific Reports. 2015;5(1). doi:10.1038/srep13185.

[17] Strona G, Lafferty KD. Environmental change makes robust ecological networks fragile. Nature Communications. 2016;7(1). doi:10.1038/ncomms12462.

[18] Burgos E, Ceva H, Perazzo RPJ, Devoto M, Medan D, Zimmermann M, et al. Why nestedness in mutualistic networks? Journal of Theoretical Biology. 2007;249(2):307–313. doi:10.1016/j.jtbi.2007.07.030.

[19] Vieira MC, Almeida-Neto M. A simple stochastic model for complex coextinctions in mutualistic networks: robustness decreases with connectance. Ecology Letters. 2014;18(2):144–152. doi:10.1111/ele.12394.

[20] Strona G, Nappo D, Boccacci F, Fattorini S, San-Miguel-Ayanz J. A fast and unbiased procedure to randomize ecological binary matrices with fixed row and column totals. Nature Communications. 2014;5(1). doi:10.1038/ncomms5114.
