# Supplementary material for: The structure and robustness of ecological networks with two interaction types
Source: PLoS Comput Biol. 2024 Jan 19;20(1):e1011770. doi: 10.1371/journal.pcbi.1011770 (PMC10830016; doi:10.1371/journal.pcbi.1011770)
Supplement: Attachment — Submitted filename: Response_PlosCBiolTripartite.pdf [file pcbi.1011770.s002.pdf]

Manuscript PCOMPBIOL-D-23-00025
'The structure and robustness of ecological networks with two interaction types'
by Virginia Domínguez-García and Sonia Kéfi

Dear Editor,

Thank you very much for giving us the opportunity to revise our manuscript 'The structure and robustness of ecological networks with two interaction types' (PCOMPBIOL-D-23-00025). We are indebted to the referees for their insightful suggestions. We considered all their comments, which were very useful to clarify the meaning and the significance of our work.

In our revised version, we have:

- Clarified the network science terminology used to work with the ecological networks in our study.
- Clarified definitions of shared set, connector nodes, and included them at the start of results to avoid making the reader return to the methods section.
- Clarified how the ranking of importance and the importance values of plants are related to each other.
- Clarified that the threshold choice to determine the proportion of shared set hubs that are connectors does not change the results.
- Clarified that in the main manuscript we focus only on random plant extinction.
- Added more information on the null models, and the results we obtained to the main text.
- Implemented a stochastic version of the co-extinction algorithm that we used on the weighted version of the mutualistic-antagonistic networks (when available) to prove that the main results hold in both cases, and also that the classical co-extinction scenario is a lower bound to the damage the ecological community can suffer.
- Start the results with an overview of the database we use, and provide more information about it.
- Corrected typos, and errors pointed out by the referees.

We believe that these changes have greatly improved the manuscript, and we hope it is now ready for publication in your journal.

Please find below a point-by-point reply to the reviewer's comments. Note that the reference lines are numbered according to the revised marked-up version, and not to the final clean version.

Yours faithfully,

Virginia Domínguez-García and Sonia Kéfi

RESPONSE TO THE REFEREES

Please note italics refer to the text of the reviewer's comments

Reviewer #1

In this paper the authors investigate the robustness of tripartite networks with two interaction types. They take the most naive approach of plant removal and consequent cascading or simultaneous co-extinction of animals in the two layers. This approach has many assumptions and limitations (e.g., no rewiring, no weights). However, as the authors claim, it serves as a starting point for basic insights into community robustness. I agree with that notion. The robustness of mulitpartite networks has already been investigated. However, this is the first work to do so in a systematic manner, and comparing three types of networks. In that sense this work is novel and is a necessary contribution to the knowledge on community robustness. There are several conceptual insights arising from this work. First, that the robustness of the tripartite networks can be predicted from that of its two constituting layers. Second, the proportion and role of connector species in the different kinds of networks. Third, that interdependent communities were not less robust to plant losses. The analysis is strong and covers multiple aspects of the structure-stability (or structure-robustness) link. The comparison to null models makes the analysis more robust and insightful, although this part is not as emphasized in the main text (see my comments).
Overall, I find this paper merits publication in PLOS Comp Biol. I have just a few comments and suggestions. Thanks for the efforts put into that work.

We thank Reviewer #1 for her/his positive assessment of our work.

1.1 The results presented in the main text are for random removal order. This is very null-model-like. It would be good to include some of the DD and ID results in the main text. Fig S9 states quite the obvious, so maybe not this one. But are there any meaningful insights regarding the effect of structural properties on robustness for nonrandom removal?

We thank the reviewer for this suggestion. We now added some text mentioning the other removal scenarios in the revised manuscript (L-159 of the revised marked-up manuscript). However, since part of the results in the manuscript can only be obtained in a random removal scenario (as explained in more detail in point 3.16 below), and since the results are complex enough, we decided to not put the figures about the other scenarios in the main text, ”but we added references to the corresponding figures (which are in SI) in the main text L-161) In line with other referee's comments, we now clarify at the start of the manuscript that we focus on random removal (L-82) and leave the more detailed results on other extinction scenarios in the supplementary (L-307).

1.2 The null models provide important insights but are hidden in the SI. I really liked the approach of increasing constraints and the figure that describes the null models. Consider moving them to the main text. I especially liked the results of interdependence that are in the SI (paragraph starting with: "Comparison with the four null models indicates that, for AA networks. . . "). Also Fig. S7, and S11.

We now refer to the null models in the introduction and clearly point to their description in the Methods section of the main text (L-93). We also included the figure describing the 4 null models in the methods. Since we already had most of the results in SI 5 and 6 in the main text, we only included those that where missing. To make the results more clear and facilitate access to the reader, we now also point directly to the panels in Fig. S7 where the results can be viewed (L-138, L-144, L-147, L-167). We decided to not include more figures in the main text because that would mean including 8 more panels, that do not provide much

visual help, and could obscure what we think are the main results of this study.

1.3 Plant importance ranking: What are the actual differences between the rankings? A plant can rank higher than another but the actual difference in importance value can be very small. What is the distribution of importance values?

We thank the referee for pointing this out. The histogram of importance of plants indicates that the ranking is particularly well defined in the case of the more important plants, both in the particular case that we use as an example in the main text (Fig R.1) as when we consider all tripartite networks together (Fig R.2). While it is more difficult to differentiate between the less important plants (i.e. those with lower importance values have importance values that are more similar), the ranking is still well defined, as shown by the correlation values between the importance and the ranking based on importance (see lower row of Fig. R.1). Also, the whole community ranking is more spread than any of the two other rankings alone, indicating that considering both interactions simultaneously can help to better gauge the importance of plants. We have included this analysis in the supplementary information (SI 7, Figures S16 and S17) and briefly comment this on L-186 in the main text.

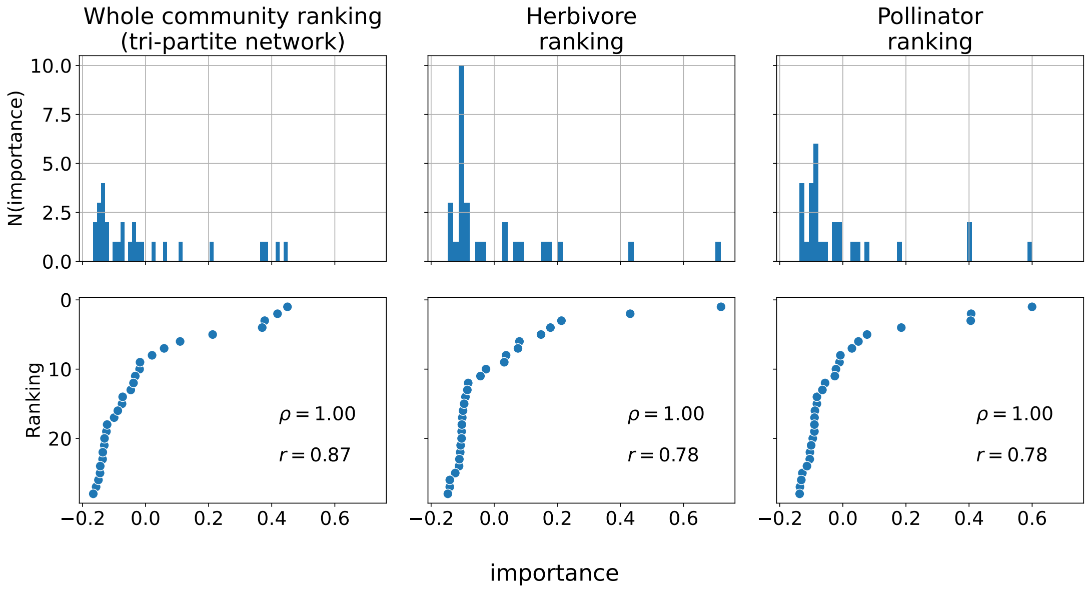

Figure R. 1: Upper row: Distribution of plant importance values in the network used as example in Fig. 4. The histograms depict the amount of plants with a given importance in the whole community (left), with a given importance for pollinators (center) and for herbivores (right). Lower row: Ranking of importance (based on importance values) vs importance. Insert texts display the Spearman rank correlation coefficient (ρ) and the Pearson correlation coefficient (r).

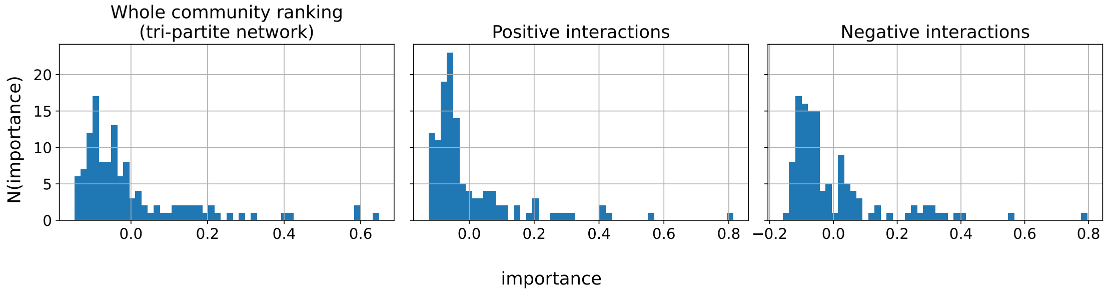

Figure R. 2: Distribution of plant importance values considering all the networks in the data-set together. The histograms depict the amount of plants with a given importance in the whole community (left), with a given importance for animals connected through positive interactions - pollination or seed dispersion - (center) and for animals connected through negative interactions - herbivory or parasitism - (right).

1.4 The inherent difference between AA and MA/MM in the role of plants will cause a "slower" (i.e., more removal events needed) extinction in AA networks. Generally, if you take a network and remove nodes from the linking set it will collapse faster than if you remove from any of the other two sets. Hence, given an AA and an MM/MA network with a similar number of nodes in corresponding sets and similar degree distributions, AA networks will be more robust than MM and MA networks. Is this the underlying cause for the differences between AA/MM-MA networks? In Fig 3B you find a pattern opposite to what I expected, whereby AA networks are less robust than MM. Why? I somehow feel that you might have explained all that in the text and I missed it...

We believe that there is no a-priory reasoning to expect that either AA or MM networks should be more robust. For example, a MM network that has very few connections between the two interaction layers can have a slower extinction than an AA network formed by many trophic chains, where losing a plant would mean losing many species up the chain instantaneously. What we found was that the robustness of the tripartite networks was overall very similar across network types (L-156), with mutualistic-mutualistic networks being slightly more robust than antagonistic-antagonistic networks, but not statistically significant. The underlying differences between robustness depend more on the particular structure of each network than on a general building principle, that is why we do not see big differences among network types, but we see significant correlations between robustness and degree distributions or the number of connector nodes (Table 1). The most interesting result of this part, we believe, is the fact that networks that are more interdependent are not necessarily less robust. Robustness is rather a consequence of the degree heterogeneity and connectance (in line with previous results in networks with only one interaction type) (L-159, L-167).

1.5 Related — L 134: Isn't the robustness of AA lower than MM? At least as far as I can tell from Fig 3B.

Yes, indeed it is. We thank the referee for pointing out this error. This was corrected in L-154 of the revised manuscript.

1.6. It was difficult to understand the results without referring to the methods to see the definitions of the structural properties. Maybe move some text from the methods to the results.

Following this suggestion, we added some explanations in the results to clarify the text and make it more accessible to the reader; please see lines L102-111, L-144 in the revised manuscript.

1.7 How sensitive are the results for the thresholds? E.g., the 20% of the .

The results were not affected by the threshold used to assess whether the shared nodes with highest degree were the ones connecting the two interaction layers or not (Fig. R.3). In the main text, we used a 20% threshold to ensure that all networks had at least 1 "most connected" node (note that the minimum possible amount of connector nodes in the shred set is 5). We now comment briefly on this in methods (L-266) and SI (Fig. S6B).

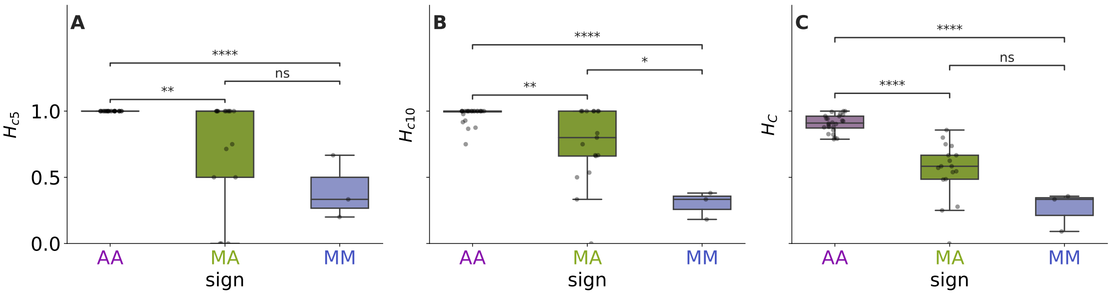

Figure R. 3: Effect of the threshold considered in determining the proportion of shared set hubs that are connector nodes (H_C), i.e. the proportion of connector nodes inside the 5% (A), 10% (B) or 20% (C) of most connected shared set nodes.

1.8 L108: change 'behaviour' to 'structure'

Done, L-122 of the revised manuscript.

1.9. Fig. 3: What are the gray data points in panels A-B? What are the green and blue points in C?

Updated legend to clarify these points.

Reviewer #2

This is a study on the robustness of multilayer networks, using as a model different kinds of tripartite networks that combine mutualisms and antagonisms. Overall, its rationale is very interesting and the topic, timely. As claimed by the authors, multilayer networks are a new frontier in Ecology, as this approach finally provided us with a tool set to address two or more interaction types together in the same system. This is a great leap forward, as interaction types do not occur isolated in nature, but rather interact with one another, generating conditional outcomes. Tripartite networks are indeed a good starting point to advance this approach also to assess the topic of stability. The study is well designed and well presented. Its language is strongly focused on the analyses and math, which would be problematic for a strictly biological journal, but is not the case in a more computational venue. After a few adjustments this study can become a highly relevant contribution to the fields of network ecology and species interactions.

We thank Reviewer #2 for her/his positive assessment of our work.

2.1 Be careful with typos and grammar errors, especially errors of number agreement. A review by a professional proofreader (native speaker or not) is always a good idea.

We carefully corrected the typos, and we asked a colleague to proofread the final version of the text.

2.2 Avoid using so many acronyms and abbreviations in the text, especially custom-made ones that are not regularly used in the field. They may shorten the text, but severely hinder its readability.

We decreased the use of all acronyms and abbreviations through the manuscript.

2.3 A central point when studying multilayer networks is the definition of the interlayer edges. Paying attention to them is the main difference between using a monolayer or multilayer approach. In the case of tripartite networks, which fit the category of "diagonally coupled", interlayer edges occur between shared species, which are present in both layers (plants, for that matter). It is important to explain those definitions more clearly and how they affect the interdependence between layers. See:
- Mucha, P. J., Richardson, T., Macon, K., Porter, M. A., & Onnela, J.-P. (2010). Community Structure in Time-Dependent, Multiscale, and Multiplex Networks. Science, 328(5980), 876 LP – 878.
- Pilosof, S., Porter, M. A., Pascual, M., & Kéfi, S. (2017). The multilayer nature of ecological networks. Nature Ecology & Evolution, 1(4), 0101.

As the referee correctly points out, our tripartite networks are diagonally coupled. However, in our case, we do not have an explicit treatment of the interlinks because corresponding nodes in different layers indicate the same identity (i.e. are the same entity), as happens in multiplex networks without interlinks[1]. We now point this out when we first describe them, in L70-73. This identity is reflected, for example, in the fact that when a plant goes extinct in an interaction layer, it also goes immediately extinct in the other interaction layer.

2.4. Please explain more clearly how you defined the "connector nodes" and "connector hubs". There is much confusion in terminology between graph theory and network theory, also when it comes to centrality. When network ecology, this confusion becomes a real mess. For instance, in mathematical studies about multilayer networks, a node that connects two or more layers is usually named a "state node". When two nodes are connected to one another in two or more layers, their connection is named a "multilink". In the field of ecological networks, those kinds of nodes and links receive other nicknames. Furthermore, connectors and hubs are usually defined in ecological studies according to two centrality metrics: participation coefficient and within-module degree. See:
- Guimerà, R., & Amaral, L. A. N. (2005). Cartography of complex networks: modules and universal roles. Journal of Statistical Mechanics: Theory and Experiment, P02001.
- Olesen, J. M., Bascompte, J., Dupont, Y. L., & Jordano, P. (2007). The modularity of pollination networks. Proceedings of the National Academy of Sciences, 104(50), 19891–19896.
- Boccaletti, S., Bianconi, G., Criado, R., del Genio, C. I., Gómez-Gardeñes, J., Romance, M., Sendiña-Nadal, I., Wang, Z., & Zanin, M. (2014). The structure and dynamics of multilayer networks. Physics Reports, 544(1), 1–122.
- Kivela, M., Arenas, A., Barthelemy, M., Gleeson, J. P., Moreno, Y., & Porter, M. A. (2014). Multilayer networks. Journal of Complex Networks, 2(3), 203–271.

We thank the referee for pointing out this potential source of confusion. Here, as we also comment to Referee#3, we decided to adopt the network science terminology (which we believe is a nice way of attempting at homogenizing terminology, not only in ecology but also across other fields). However, in network science terminology there is no consensus on how the shared sets between interaction layers, nor the nodes with connections in both interaction layers, should be referred to.

We decided to change the *"linking set"* to *"shared set"* (L-68), as the set of species that may have

interactions in the two interaction layers (e.g. plants in a mutualistic-antagonistic network) to avoid using the term "link" in there, which could lead to confusion with interlinks.

We defined *"connector nodes"* as those nodes in the shared set that actually have links in the two interaction layers (e.g. a plant that is pollinated and eaten). We now define them at the introduction (L-69), and repeat it at the start of the results (L-108).

We agree with the referee that the term "connector hub" that we previously used in the methods section was confusing. We now clearly refer to these nodes only as "the proportion of shared set hubs that are connector nodes" (L-108 and 265). As previously reported to Referee#1, we now also clarify that the threshold used to determine the proportion of shared set species that are considered hubs does not affect the results (L-266).

As suggested by the referee, We have now changed the term "participation ratio" to "participation coefficient", in accordance with the literature we cited.

2.5. In this study, the classical "Memmott approach" (or Barabasi-Albert) was used to assess secondary extinctions. As commented by the authors themselves, this approach is very simplistic and unrealistic, although it was a good first step in the early 2000s, when studies on the robustness of ecological networks began to appear. Nevertheless, much more realistic approaches have been proposed in recent years, which should be also considered. See: -Vieira, M. C., & Almeida-Neto, M. (2015). A simple stochastic model for complex coextinctions in mutualistic networks: robustness decreases with connectance. Ecology Letters, 18(2), 144–152.

Following the reviewer's suggestion, we implemented the stochastic version of the co-extinction algorithm in mutualistic-antagonistic networks. Below we show the comparison between our results in the main text (using the classical approach) and those obtained with the stochastic model on weighted networks, when available. When weights were not provided in the data, we considered all weights equal to 1, which still allows to implement the stochastic version of the co-extinction model.

Fig R.4 shows that the values of Robustness (left) and Interdependence (right) are similar independently of the algorithm used, in particular for Interdependence they are almost identical. Note that in the left figure, the values on the horizontal axis (Robustness) are always higher than their stochastic weighted counterparts in the vertical axis, which corroborates that the classical approach is more conservative at quantifying robustness, providing a lower bound.

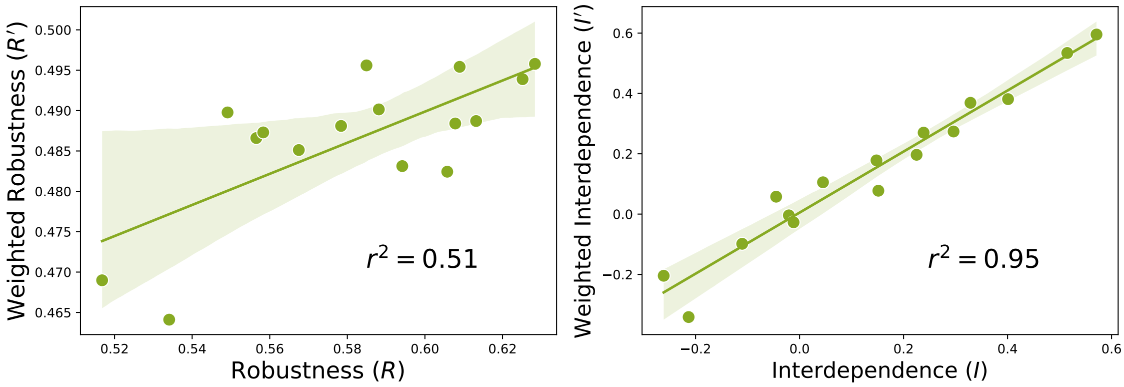

Figure R. 4: Comparative of results between the stochastic co-extinction algorithm in weighted networks (when available) with the results of the classic co-extinction algorithm used in the main text, for Robustness (left) and Interdependence (right).

Fig R. 5 shows that the Robustness of the merged network is a combination of the robustness of larger and smallest interaction layers also when using the stochastic weighted version of the co-extinction algorithm.

In light of these results, we decided to keep only the most parsimonious co-extinction algorithm in the main text, since it represents a lower bound with which compare the robustness of the communities and does not depend on any parameter. We, however, included the stochastic algorithm in the provided code, we added these results showing that the classical approach represents a lower bound to the damage suffered by the community in the supplementary information (SI 6, Fig S14 ans S15) and we comment on it briefly in the main text L-173, and methods L308-313.

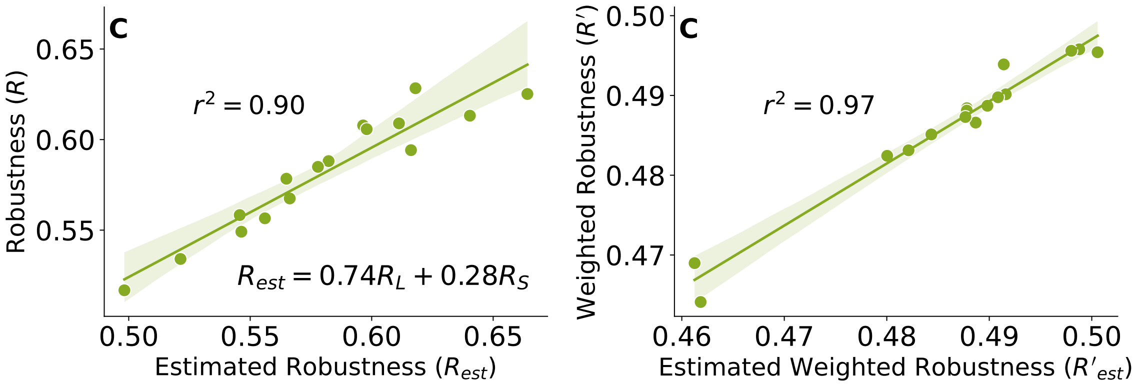

Figure R. 5: Comparison of the estimated robustness of the mutualistic-antagonistic tripartite networks as a combination of the robustness of the larger and smaller bipartite networks using the basic algorithm (left, as in the main text) and using the stochastic version of the algorithm and weighted networks (right).

2.6. The authors proposed some new null models to investigate the topology of tripartite networks. This is a

very nice step forward in the field of multilayer networks, especially in Ecology, where null model analysis is virtually considered mandatory. However, caution is advised. Recent studies have pointed out that some null models have been used wrongly in ecological studies. In spite of removing some undesired processes from network assembly, they might be actually simulating those same processes. This is the case of some of the null models used in this study, which were derived from classical null models proposed by Bascompte and Vázquez, among others. See:
-Dormann, C. F., Frund, J., Bluthgen, N., & Gruber, B. (2009). Indices, Graphs and Null Models: Analyzing Bipartite Ecological Networks. The Open Ecology Journal, 2(1), 7–24.
- Farine, D. R. (2017). A guide to null models for animal social network analysis. Methods in Ecology and Evolution, 8(10), 1309–1320.
- Pinheiro, R. B. P., Dormann, C. F., Felix, G. M., & Mello, M. A. R. (2021). A novel perspective on the meaning of nestedness with conceptual and methodological solutions. BioRxiv, 2021.04.05.438470.

We thank the referee for pointing out the importance of choosing null models appropriately and for sharing those references. In this work, we not aim to claim that any particular ecological process is behind the emergence of the structures we find in empirical networks. Rather, our idea was to build null models with increasing constraints in order to see to what extent the additional structural constraints could contribute to explain the network's robustness and interdependence. We now specifically note this on L-93. We are aware of the weaknesses of one of the null-models we included, which is why we used a new method to sample the more restrictive null model [2] and ensure an unbiased sampling of that random ensemble.

2.7. Isn't there a better way to visualize tripartite networks? Yes, on the one hand, they are traditionally plotted as two bipartite graphs connected to one another. On the other hand, those "bipartite drawings" are quite poor in depicting topology and centrality. They serve only the purpose of visualizing nestedness. Consider more efficient, imaginative designs. See:
- Pocock, M. J. O., Evans, D. M., Fontaine, C., Harvey, M., Julliard, R., McLaughlin, Ó., Silvertown, J., Tamaddoni-Nezhad, A., White, P. C. L., & Bohan, D. A. (2016). The Visualisation of Ecological Networks, and Their Use as a Tool for Engagement, Advocacy and Management. In G. Woodward & D. A. Bohan (Eds.), Advances in Ecological Research (1st ed., pp. 41–85). Academic Press.
- Marai, G. E., Pinaud, B., Bühler, K., Lex, A., & Morris, J. H. (2019). Ten simple rules to create biological network figures for communication. PLOS Computational Biology, 15(9), e1007244.

The referee is right that there are many ways to represent networks with multiple interactions that may be more appealing to the eye. Here, we nonetheless decided to keep the representation we have of the tripartite networks in Figure 1. The reason is that we believe that the new, circular, representations of the networks would not be more informative that the figure we already had. Indeed, while this representation is quite classical, it clearly shows what we wanted to portray: the differential connection patterns in the shared nodes in different network types. Note that that first figure is a figure to introduce the concepts and terms and not a result figure, so a simpler representation seemed more efficient to us.

Reviewer #3

Overview: I reviewed this manuscript with great interest. As the authors point out, it is only recently that datasets of networks comprised of multiple interaction types have been published in high enough numbers to start drawing conclusions across them. Here, the authors examine networks consisting of multiple antagonistic interactions, multiple mutualistic interactions and a combination of mutualistic and antagonistic interactions. They investigate structural differences and similarities as well as how robustness changes among

these types of networks, and the possibility of emergent properties in the whole network not captured in the component webs. The analyses are for the most part sound and conclusions are broadly well-supported. However, I do have some questions about the effects of some analytical decisions (detailed below). For instance, it would be helpful for the authors to comment on how collection/rearing methods might be contributing to the conclusions. Also, how the decision to use unweighted networks might affect the outcome; a lot of information is lost by removing interaction strength so perhaps the authors can comment on how they might expect their conclusions to change with weighted networks? Some more technical aspects of the network metrics and descriptions could be explained earlier and in more detail (e.g. degree, connectors and hubs) to help non-specialist understanding. It is generally well written (although I have included some typos and suggestions for rewording a few awkward phrases at the end). I detail some further questions and suggestions for improvement below, but in general, I found it to be an interesting read that presents novel cross-study conclusions.

General comments: I tend to think of tripartite networks to mean three sequentially linked trophic levels (e.g. plant-herbivore-parasitoid, or plant-herbivore-consumer), rather than combining networks of 2 interaction types that share a common resource (e.g. plant-pollinator + plant-herbivore). There's nothing inherently incorrect with your definition, but to me it confuses the terminology a bit in a field that already has multiple terms for the same thing. Please consider using another term to describe combining networks of different interaction types. Annoyingly, the field is far from unanimous in what to call this type of network, but perhaps consider something like: network of networks, network of multiple interaction types, multilayer network, combined network, hybrid network... A paper that might have been missed that also examined robustness across networks of different interaction types is Morrison et al. (2020). I am not suggesting this be added to the analyses per say, but it might be of interest to the authors.

We thank the reviewer for pointing out this potential confusion in the terminology. Here, we strongly base ourselves on the network science terminology (which we believe is a nice way of attempting at homogenizing terminology, not only in ecology but also across other fields). For that reason, we now more specifically define terms in the paper: following comment from Reviewer#2 we now clarify that our networks are multilayer diagonally coupled networks without interlinks[1], and that we deal with a particular case of those which is referred to as tripartite throughout the manuscript (L-70). Tripartite (or more generally k-partite networks) are a type of multilayer networks with both coloured nodes an coloured edges, [3], a term that has been previously used in the ecological literature to refer to the type of ecological networks with two bipartite interactions layers that we study here [4, 5].

We also thanks the reviewer for pointing out this interesting reference. We have now included it in the introduction, when we present the advances that have been made in the study of the structure and robustness of networks including more than one ecological interaction (L-55).

3.1 1 Line 76: In what order were species removed? Random? By degree? Can you clarify here?

We now clarify that we study random removal in the beginning of the manuscript (L-78).

3.2 Line 85: Your methods would only highlight keystone plant species, correct? Not a particularly well connected/central insect?

Correct, we now specify it in L-92.

3.3 Lines 87-91: It reads a bit strangely to have the conclusion of the results in the introduction before having presented the results. Consider moving this to the discussion, and rather in this paragraph, outlining

specifically which hypotheses you were testing/investigating and predictions.

We concluded the introduction in this style because it is included in the journal guidelines: *"The Introduction should conclude with a brief statement of the overall aim of the experiments and a comment about whether that aim was achieved"*.

3.4 Line 92: It would really help with readability to first have a paragraph summarising the datasets used and key properties of them (interaction types, size etc.).

We now start the results section with a brief description of the database, L102-105 of the revised marked-up manuscript.

3.5 Line 95: The Connector is a node that exists in both networks, correct? Not necessarily one with a particular property (e.g. high betweeness)? This could be made clearer at this point to ease understanding

Agreed. We clarified this point in text, L-108 of the revised manuscript.

3.6 Line 100: How many linking set hubs were there?

The number of connector nodes in the tripartite networks we studied ranges from 5 to ~ 100, while the number of nodes in the shared set (before known as linking set) ranges from 14 to ~ 270 (see the modified version of Table S2 that now include sizes). To be able to compare between networks of different size, we considered the proportion of shared set hubs (i.e. the 20% of nodes in the shared set with most connections) that are connectors, rather than the raw number. The rationale is that the proportion will always be within 0 and 1 in all networks, while the raw number can be more variable. We fixed the 20% threshold for convenience, but as shown in point 1.7 the threshold choice does not have a important impact in the results (see Fig. R.3).

3.7 Line 119-120: How much of the interconnection in AA networks is due to it being parasitoids reared out of herbivores? Leaves with herbivores are collected and parasitoids are coming from them so they will, by necessity, be strongly interconnected. It is not necessarily the antagonism but the specific mechanism or sampling design.

We cannot answer this question as we do not have independent samplings of the parasitoids, we only have the information provided by the different studies we gathered. In these studies parasitoids are frequently obtained from the herbivores on leaves, as the referee correctly points out. We can not be sure about the extent to which the correlation we find is because there is a strong dependency between parasites and hosts (since one cannot live without the other) or if it is a consequence of the sampling method. With the information that we have, we find that AA networks (i.e. food-web like structures) are more interdependent than the other two types, but it is due to their basic structure, since we also find that the empirical AA networks are not more interdependent than any of their randomizations. Elucidating the issue suggested by the referee is out of the scope of our paper (as we do not have the means to tackle the problem), but we now mention this possible issue in the results (L-135).

3.8 Line 165: How much does this trend for dissimilarity between the sizes of networks affect your other findings? I suspect some of the MA networks were also pollinator dominated, no? Could this have skewed more of your findings?

In terms of Robustness, the difference of size seems not to be too relevant. In Fig S11 for example, we show how the Robustness of the merged network can be obtained as a combination of the robustness

of the larger and smaller bipartite networks, and this works for all networks, including those with more size differences. In general, mutualistic-antagonistic networks where not more dissimilar in sizes than antagonistic-antagonistic networks (see Fig. R.6). The only ones showing a high disparity in sizes were those including two types of mutualism (pollination and seed dispersal, or pollination and ant mutualism), and this trend is reduced when one considers the relative size dissimilarity, rather than just the raw value. Hence, we do not think that many of the results should be skewed because of size dissimilarity in networks. We now include the size of the two interaction layers in Table S3.

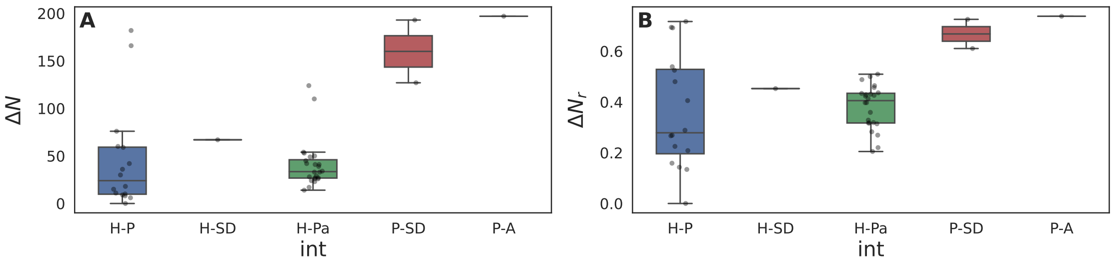

Figure R. 6: Size dissimilarity between the largest and smaller interaction layer, grouped by the different interaction layers composing the tripartite network: H-P (hervibory - pollination), H-SD (herbivory - seed dispersal), H-Pa (Hervibory parasitism), P-SA (pollination - seed dispersal), and P-A (pollination - ant mutualism) considering A) raw size dissimilarity (number of species in largest interaction layer - number of species in smaller interaction layer), and B) relative size dissimilarity (raw size dissimilarity divided by the number of species in the largest interaction layer).

3.9 Line 183: Can you test for the effect of abundance? Or control for it in your analyses?

Most of the data-sets in this study do not have information about species abundances, which makes it impossible to control for it.

3.10 Line 206-7: Your analyses don't account for any species that might exist in multiple networks (e.g. butterflies). Can you comment on how this might affect your conclusions? Could the impact of looking at robustness in combined networks be stronger if this were included?

We did not find any such case in the databases we used. In them, animal species where found only in one set at a time. As a though exercise, following the reviewer's suggestion, it could be possible to consider these species in different ways, for example including them twice (as pollinator and as herbivore). We believe that the way in which these species are included may have an effect in the results, but we are not sure this effect is easy to predict. For example, it could be that including a species twice will automatically translate in reduced robustness, because by killing one plant we would record twice the extinctions if the species depends on the same plants in the two interaction layers. One should note, however, that if the species depends on completely different partners in the two interaction layers, the effect would be the contrary, and we could find that the network is more robust. Hence, we believe this is not easily solved until one has field information on how "overlapping" are the species partner's in both interaction layers.

3.11 Line 224: Using unweighted networks means potentially important information is lost when removing interaction strength. Can you comment on how this might have affected your conclusions? Was there any attempt to normalise across networks of different sizes?

Following Reviewer#2's suggestion, we implemented a stochastic algorithm using weighted networks (when available) where the probability of species co-extinction increased with its dependence on the species that had undergone extinction. As can be seen in Figs R.4 and R.5 the unweighted classical approach to quantify robustness sets a lower bound on the damage the ecological community sustains, and the results we obtained using the classical approach hold when we use the stochastic and weighted method. In light of this, while considering interaction weights has important consequences for the dynamics of the ecological community, it seems not to be very relevant for the approaches based on network topology. As explained in response 2.5 to Reviewer #2, we decided to keep only the most parsimonious co-extinction algorithm in the main text, since it represents a lower bound to the damage the community might suffer, and does not depend on any parameter. However, we added the stochastic algorithm to the provided code, and we included the results showing that the classical approach represents a lower bound in the supplementary information (SI 6, Fig S14 ans S15) and mentioned it briefly in the main text L-173 and methods L-308.

3.12 Line 235: Non-specialists might not know what degree is. Probably worth defining on first use.

Agreed, included a brief definition when degree is first mentioned in the main text (L-144) an in Methods (L-265).

3.13 Line 243: How was the order of removal determined? From least connected/lowest degree to most? randomly? another way?

We now clarify from the beginning that in the manuscript we focus on random removal only (L-82).

3.14 Line 256: Multipartite networks is inconsistent terminology. I say above about my preference to not use tripartite here, but whatever term is used, it should be consistent

We changed the word "multipartite" to "tripartite" in L-203, to avoid inconsistent terminology. With the use of "multipartite" in L-289, we are referring to the two networks presented in the studies that are cited in that sentence, and that are composed by multiple bipartite networks (and not only by two, as those in our study). We left the word multipartite here to refer to them, but clarified that we are referring to those networks, and not to the tripartite networks in our study (L-289 of the revised manuscript).

3.15 Line 271: So the extinction sequence is based on random removal? Can you clarify this earlier

Agreed, we clarify it now at the start of the manuscript (L-82).

3.16 Line 281-2: This is a neat approach. Does it correlate at all with degree? Did you get the same/similar importance when species were removed in order of degree?

The approach we used to determine plant importance based on the impact their extinction has on the community relies on their order in the extinction sequence. The only way to explore all the possibilities is to consider random extinction sequences, in which a given plant may appear in any ordering. That is why we (and the study we used for reference) only use random extinction sequences to determine the importance of plants based on the damage they may cause to the ecological community. Removing plant species ordered by degree means that we can explore just a subset of all the possible orderings, and hence that approach will only give information on the relative ordering of the plants that have a similar degree (and as a consequence will change its relative position on the extinction sequence), but not on all of them. In particular, in the networks in our study we find a relatively high degree heterogeneity, meaning that there are a few generalist species with high degree (that in a decreasing/increasing degree scenario will always be on the exact same position) and more specialist species with degree 1 (that will be able to change relative positions among

them). This means that carrying out the study in a decreasing/increasing degree scenario will only provide information on the relative position of these specialists species, but not on all the species, which is not what we are looking for.

As for how the ranking of plant importance correlates with node degree, while there is a trend of a positive relationship between degree and ranking of importance, the correlation values are not too high due to the high redundancy of the degree values (Fig. R.7), a result similar to what we found in the pollination and the herbivory layer. Hence, it is not possible to predict the importance of a plant for the ecological community only by knowing its degree.

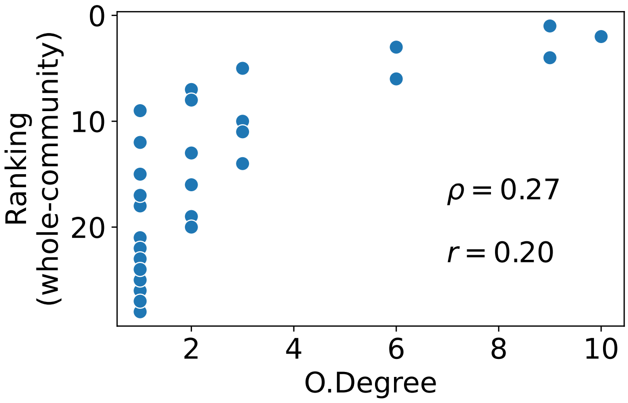

Figure R. 7: Scatter-plot of Plant importance ranking vs its total degree for the example network in Fig. 4. Included are the correlation values using Pearson correlation coefficient (r) and Spearman correlation rank (ρ)

.

3.17 SI table 1: How did you handle multiple types of herbivory (e.g. leaf miners, caterpillars)? Were they combined into 1 network? Or did you not use networks with combined herbivory (although I think some of the studies did have multiple herbivores, no?)

They were combined, yes. Now we briefly comment on this on L-258.

*3.18 Figures: Fig. 2 caption: Please define PR in the caption for clarity. The asterisks seems 1 too many. I tend to think of * for ¡ 0.05, ** for ¡ 0.01, *** for ¡ 0.001*

Indeed, there was an error in the figure captions, and the number of asterisks to indicate statistical significance are those indicated by the referee. We changed the figures accordingly. We also included the full name of the metric in figure 2 legend.

3.19 Minor issues (e.g. typos, syntax etc.): Line 27: "trough" should be "through" Line 100: "the ones doing the connection" is awkward wording. Consider something along the lines of "... are acting as connectors" or "are forming connections" SI 2: 1st paragraph "less" and "more" should be "least" and "most", respectively In NL2 paragraph (and other places): "allows to study" is not correct wording. Replace with "allows the study of" or "allows us to study" or similar.
References: Morrison, B. M. L., Brosi, B. J. & Dirzo, R. Agricultural intensification drives changes in

hybrid network robustness by modifying network structure. Ecology Letters 23, 359–369 (2020).

We thank the referee for the care full revision. We implemented all the suggested corrections.

References

[1] Bianconi G. Multilayer Networks: Structure and Function. Paperback ed. Oxford University Press; 2023. Available from: https://lead.to/amazon/com/?op=bt&la=en&cu=usd&key=0192865544.

[2] Strona G, Nappo D, Boccacci F, Fattorini S, San-Miguel-Ayanz J. A fast and unbiased procedure to randomize ecological binary matrices with fixed row and column totals. Nature Communications. 2014;5(1). doi:10.1038/ncomms5114.

[3] Kivela M, Arenas A, Barthelemy M, Gleeson JP, Moreno Y, Porter MA. Multilayer networks. Journal of Complex Networks. 2014;2(3):203–271. doi:10.1093/comnet/cnu016.

[4] Emary C, Malchow AK. Stability-instability transition in tripartite merged ecological networks. Journal of Mathematical Biology. 2022;85(3). doi:10.1007/s00285-022-01783-7.

[5] Timóteo S, Albrecht J, Rumeu B, Norte AC, Traveset A, Frost CM, et al. Tripartite networks show that keystone species can multitask. Functional Ecology. 2022;doi:10.1111/1365-2435.14206.
