# Supplementary material for: The structure and robustness of ecological networks with two interaction types
Source: PLoS Comput Biol. 2024 Jan 19;20(1):e1011770. doi: 10.1371/journal.pcbi.1011770 (PMC10830016; doi:10.1371/journal.pcbi.1011770)
Supplement: Attachment — Submitted filename: Response_2.pdf [file pcbi.1011770.s003.pdf]

Manuscript PCOMPBIOL-D-23-00025
'The structure and robustness of ecological networks with two interaction types'
by Virginia Domínguez-García and Sonia Kéfi

Dear Editor,

Thank you very much for giving us the opportunity to revise our manuscript 'The structure and robustness of ecological networks with two interaction types' (PCOMPBIOL-D-23-00025). We are indebted to the reviewers for their insightful suggestions. We believe that these changes, together with those suggested previously by all the reviewers, have improved the quality of our manuscript.

In our revised version, we have:

- Removed the sentence on tripartite networks as suggested by Reviewer #1,
- Clarified the implications that the sampling method might have for the structure of tripartite Antagonistic-Antagonistic networks.

Please find below a point-by-point reply to the reviewer's comments. Note that the reference lines correspond to the revised marked-up version, and not to the final clean version.

Yours faithfully,

Virginia Domínguez-García and Sonia Kéfi

RESPONSE TO THE REVIEWERS

Please note italics refer to the text of the reviewer's comments

Reviewer #1

The authors have addressed my comments. My only comment is that they should remove line 69-73 (starting with: "In the network science literature..."). This statement is wrong. Diagonally-coupled networks have interlayer links. The tripartite networks are not multilayer and do not have interlayer links. They are multipartite (tripartite in this case).

We are glad that the reviewer found that our analyses clarified the raised issues, and we thank her/him again for the useful comments. We have deleted the sentence on tripartite networks, as suggested.

Reviewer #2

The authors did a terrific job revising their manuscript. This new version is much better than the previous. I have no further suggestions to make.

We are happy to receive such a positive assessment of our work, and we thank the reviewer again for her/his insightful comments. We really think they helped improve the manuscript.

Reviewer #3

The authors have done a great job of addressing the comments and this revised version is much improved. It is clearer to read and the questions about the methodology have been fully addressed and the terminology and approach is much clearer throughout.

We highly appreciate the positive assessment of the revised version of our manuscript, and we are glad to hear that our analyses clarified the raised issues. Here below, we address in detail the remaining points raised by the reviewer.

The main point that remains, to my mind, is with regard to the relevance of the comparisons between different types of tipartite networks. Because the original data sets were not necessarily collected to compare between the different bipartite networks that comprise them, some of the detected differences might be due to sampling differences rather than structural ones. In particular, the antagonistic-antagonistic networks are all plant-herbivore-parasitoid networks which is a specific kind of A-A network (as supposed to say, herbivory and insectivory combinations that could be collected independently). These are typically collected by sampling a herbivore on a plant and then rearing a parasitoid out of it. Whereas a plant-pollinator/plant-herbivore or plant-pollinator/plant-seed disperser network would have 2 independent measures for each interaction. This means that the herbivore linking to a parasitoid is the only way a parasitoid could be in the network. The authors now note that this is relevant for the robustness analyses as any plant removal simulation would necessarily propagate up the A-A network due to the sampling design (but also biologically) rather than any network property (a parasitoid has no ability to interact with a plant outside of the sampled herbivores). But I think it is important for the "fundamental differences" reported as well. I'm not sure there is a way to disentangle this confounding effect, and the conclusions are still relevant and interesting, but it is a consideration that needs to be acknowledged, particularly in the discussion. Specifically, in lines

210-212: the conclusion that the hubs being connectors is because generalist herbivores might be more abundant, could also be because the parasitoid can only be reared out of one of the sampled herbivores. I would be very cautious about the interpretation here being about an ecological or structural difference when it could be an unavoidable sampling effect.

We thank the reviewer for pointing this out. This is an important point to make clear in the paper. In the previous revision, we had added clarifications in that regard in the results around L. 134. We now further clarified that point (L 135-136) and also refer to this in the discussion section (L 213).

The same is true in the respective results (lines 113-114) regarding the equal splitting of links. Sampling methods mean the herbivore hub would necessarily be equally split between the 2 layers. If the links were collected independently (as in plant-pollinator/plant-herbivore networks), then you might expect fewer shared links. It is important to distinguish which of the results are due to collection method differences, especially since the data were typically not collected to test for these sorts of differences so the variable collection methods are likely to be an important confounding effects.

Although we agree with the reviewer on the previous point, on this one we would like to argue that the fact that the links are more equally split in these networks is not necessarily a consequence of the sampling method. It could indeed very well be that generalist herbivores (that feed on many different plant species) were those having less parasites or vice-versa, which could lead to an unbalanced distribution of links between the two layers, and this regardless of the sampling method. In view of this, we decided not to edit that sentence, since it was just a mere description of the results.

Otherwise, the conclusions are well supported by the results and the approach including multiple null models and now including weighted networks, where possible, is sound and much improved!

We thank the reviewer again for her/his positive assessment of our work, and we hope that the new changes, together with the previous ones, addressed all her/his concerns.

Very minor typo/syntax suggestions: Line 74: "consists in 44…" should be "consists of 44…"

Changed accordingly in L 74.

DATA ACCESIBILITY
Have the authors made all data and (if applicable) computational code underlying the findings in their manuscript fully available? The PLOS Data policy requires authors to make all data and code underlying the findings described in their manuscript fully available without restriction, with rare exception (please refer to the Data Availability Statement in the manuscript PDF file). The data and code should be provided as part of the manuscript or its supporting information, or deposited to a public repository. For example, in addition to summary statistics, the data points behind means, medians and variance measures should be available. If there are restrictions on publicly sharing data or code —e.g. participant privacy or use of data from a third party—those must be specified.

Reviewer #1: No: I did not see a link to the data the authors used. I might have missed it, but it must appear. Referring to the original papers is not good enough because sometimes there are changes when using other peoples data. The authors should store their data online in a permanent data repository (Figshare, dryad, etc).

Reviewer #2: Yes

Reviewer #3: None

The Database and codes were available in GitHub (as stated in the data accessibility statement). We are sorry that this was not clear to some of the reviewers. The Database of networks, together with the latest codes used to generate the results shown in the manuscript and in supporting information, are now publicly available via Zenodo [1].

References

[1] Domínguez-García V, Kéfi S. Data and code corresponding to the article 'The structure and robustness of ecological networks with two interaction types'. Zenodo. 2023;doi:10.5281/zenodo.10198613.
